# REDETR-RISTD: Real-Time Long-Range Infrared Small Target Detection Network Based on the Reparameterized Efficient Detection Transformer

**DOI:** 10.3390/s25092771

**Published:** 2025-04-27

**Authors:** Ning Li, Daozhi Wei

**Affiliations:** Air Defense and Antimissile School, Air Force Engineering University, Xi’an 710051, China; 15339159188@189.cn

**Keywords:** long-distance infrared small target detection, lightweight feature extraction backbone, attention mechanism, multi-scale pyramid, bidirectional fusion

## Abstract

The critical challenge of detecting infrared small targets at long ranges is that accuracy is compromised. This happens because the targets are small in size, have a weak signal-to-noise ratio (SNR), and are surrounded by complex backgrounds. A novel real-time long-range infrared small target detection network based on the Reparameterized Efficient Detection Transformer (REDETR-RISTD) is proposed. REDETR-RISTD maintains accuracy while significantly reducing computational complexity. First, we introduce a self-developed reparameterized multi-scale feature extraction module (RMSFE). This module helps to construct the lightweight RepEMSNet backbone. It substantially reduces model parameters while maintaining detection capabilities. Second, we design an Attention-Based Intra-Scale Contextual Features Interaction (AICFI) module within the hybrid encoder. This module enhances focus on infrared small targets. It also improves feature interaction across scales. Third, we implement a multi-scale pyramid feature fusion network (MSPFN) with bidirectional fusion mechanisms. This architecture helps to better capture and enhance small target features. Experimental results across three representative public datasets demonstrate the effectiveness of our approach. Compared to state-of-the-art (SOTA) models, REDETR-RISTD has only 13.814 M parameters. It achieves competitive performance with AP50 and recall rates of 96.5% and 92.7%, 84.3% and 83.3%, and 98.5% and 97.9%, respectively. REDETR-RISTD successfully balances the trade-off between detection accuracy and computational efficiency.

## 1. Introduction

Infrared small target detection technology has emerged as a critical tool across diverse operational domains, encompassing maritime reconnaissance, aerial surveillance, emergency response, environmental observation, and clinical diagnostics. This technology’s significance derives from its exceptional concealment capabilities, resistance to interference, and consistent functionality across all environmental conditions, establishing it as a fundamental component in contemporary monitoring systems [1,2,3,4].

The application landscape of infrared imaging extends well beyond these traditional domains. In the transportation sector, pioneering work by Bertozzi et al. [5] has yielded thermal night vision systems capable of identifying roadway hazards and pedestrians during limited visibility, substantially improving vehicular safety parameters. The behavioral sciences have similarly capitalized on this technology, with Koukiou and Anastassopoulos [6] establishing that thermal facial signatures provide reliable indicators of alcohol consumption, while subsequent research by Koukiou [7] has expanded these methodologies to broader cognitive state evaluation. Ecological applications have incorporated infrared systems for fauna monitoring and conservation initiatives, with particular emphasis on early thermal detection of forest conflagrations as elucidated by Pastor [8]. In healthcare contexts, Ring and Ammer [9] have documented the effectiveness of infrared imaging in non-invasive identification of inflammatory conditions and vascular irregularities. Currently, recent innovations documented by Koukiou [10] have advanced biometric security protocols through thermal facial vasculature mapping. This expansive functional spectrum underscores infrared imaging’s transformative capacity across scientific and practical domains.

**Figure 1:**
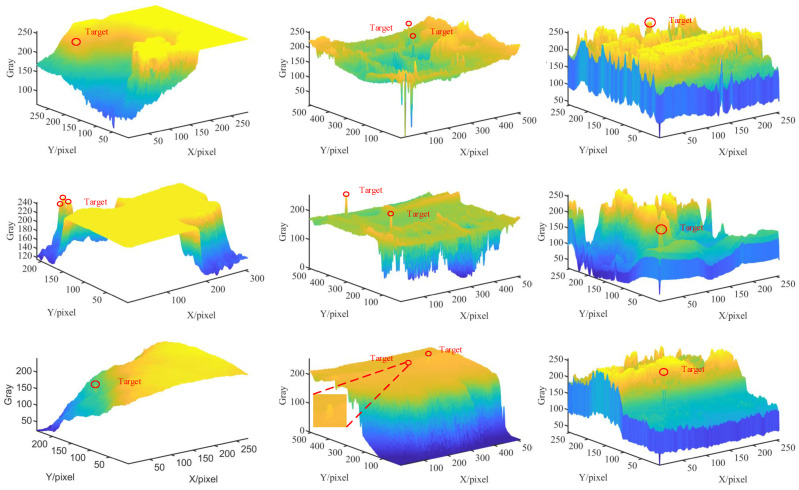
Typical infrared small targets. The red circles indicate the locations of the small targets.

The increasing requirement for precision in infrared small target detection has necessitated algorithmic advancement to overcome inherent limitations including diminutive target dimensions, suboptimal signal-to-noise ratios, and complex background interference [1,2]. As illustrated in Figure 1, infrared targets typically manifest as minimal, low-intensity signatures with substantial background similarity. Consequently, this investigation explores sophisticated infrared small target detection algorithms, with the objective of developing optimized methodologies that enhance detection precision, minimize false positive indicators, and improve infrared system efficacy across practical implementation scenarios.

Traditional infrared small target detection methods are mostly model-driven and include filtering-based, local contrast-based, and tensor-based approaches. For example, Lizhen Deng et al. used quantum genetic algorithms to enhance the top-hat filter for adaptive detection [11], and Yi Cui et al. combined heterogeneity filter convolution with hollow side window filtering and patch contrast measures for precise target identification [12]. These methods have been effective in certain specific applications but often require integration with additional techniques to optimize performance. X Zhang et al. and Zhonghua Wang combined various filtering and contrast mechanisms to differentiate targets from backgrounds and reduce false alarms [13,14], while Yongsong Li et al. and Xiangyue Zhang et al. introduced new filtering methods and multi-scale templates to improve detection in complex backgrounds [15,16].

However, these methods have limitations in complex scenes, particularly when there is significant noise and clutter, which reduces robustness. The complex background often complicates the extraction and utilization of local contrast information, and slight fluctuations in hyperparameters can significantly impact detection performance, limiting their generalization ability.

In recent years, deep learning, especially convolutional neural network (CNN)-based methods, has brought significant breakthroughs in infrared small target detection. For instance, Yu et al. proposed attention mechanisms that effectively guide shallow features to deep features, enhancing feature fusion [17], while Tong et al. introduced the EA-ATT module to improve the interaction between deep and shallow features [18]. Li et al. introduced the DNANet network, which uses densely nested attention networks to progressively fuse features [19]. These methods have improved detection accuracy but still face challenges, especially in long-range infrared small target detection, due to small target sizes, low contrast, and high background complexity [20].

Compared to traditional methods, deep learning-based techniques directly learn features from the raw image, offering stronger adaptability and generalization capabilities [20,21]. For example, Wang et al. and Zhao et al. employed generative adversarial networks (GANs) to balance missed detection and false alarm rates, focusing on noise suppression and target feature enhancement [21,22], while Zhou et al. proposed a competitive framework that achieves a Nash equilibrium between false negatives and false positives [23]. Additionally, Zhao et al.’s TBC-Net [24] and Hu et al.’s ST-Net [25] contributed significantly to small target detection by introducing residual connections and smoothing propagation.

Despite the significant improvement in accuracy, these deep learning-based methods still face the challenge of balancing real-time detection with high precision, especially for small targets. Therefore, we propose REDETR-RISTD, a single-stage infrared small target detection network based on the Reparameterized Efficient Detection Transformer. REDETR-RISTD combines the strengths of existing methods and optimizes them to enhance detection accuracy while maintaining high real-time performance.

The main contributions of this paper are summarized as follows:

(1) We propose a single-stage infrared small target detection method, REDETR-RISTD, which achieves real-time, high-precision detection in complex backgrounds and significantly reduces missed detections.

(2) We introduce a lightweight backbone network, RepEMSNet, built upon the RMSFE module. RepEMSNet enhances multi-scale feature extraction, improving the receptive field for small targets while reducing inference time through structural reparameterization.

(3) In the encoder, we use the AICFI module to capture salient features of infrared small targets and employ MSPFN for bottom-up feature fusion and top-down feature enhancement, thereby boosting the network’s detection ability.

(4) Experimental results demonstrate that REDETR-RISTD outperforms other SOTA detectors on three datasets, showing vast potential for applications in remote sensing and early warning detection.

## 2. Related Work

### 2.1. Vision Transformer (ViT)

Dosovitskiy et al. [26] first applied the Transformer [27] to computer vision by introducing the vanilla Vision Transformer (ViT). However, when applied to broader computer vision tasks such as object detection and semantic segmentation, vanilla ViT encountered several challenges. In particular, it struggled to capture local feature information and achieve precise target localization, required extensive data for training, and had a complex, computationally expensive structure.

To overcome these limitations, subsequent research has focused on enhancing ViT’s capabilities. Beal et al. [28] adopted a hybrid approach by integrating ViT with CNNs to create ViT-FRCNN, which leverages the spatial feature maps generated by ViT within the Faster R-CNN framework to improve detection efficiency. Fang et al. [29] further extended this idea by proposing YOLOS, a series of ViT-based object detection models, demonstrating the versatility of pre-trained Transformers in object detection through modifications in label and loss functions.

Li et al. [30] introduced complex masked image modeling (MIM) pre-training to boost ViT’s performance in object detection. Building on this, Fang et al. [31] proposed MIMDET, which utilizes MIM pre-trained ViT, replaces patchify stems with compact convolutional stems, and generates multi-scale feature maps from a single-scale ViT, leading to significantly faster convergence. In another line of work, Lou et al. [32] focused on multi-scale ViT to enhance defect target detection accuracy on printed circuit boards, achieving high detection accuracy with their approach.

Other contributions include Wang et al. [33], who tailored a visual transformer detector (ViTDet) for aerial image object detection and outperformed advanced CNNs on multiple datasets. In contrast, Motional et al. [34] argued that deeper models do not necessarily yield better results in 3D object detection and thus developed efficient network training strategies to reduce inference time while maintaining high performance. Zhang [35] proposed an efficient inductive visual Transformer framework and validated its effectiveness in optical remote sensing image detection tasks.

More recently, Li et al. [36] introduced the pyramid convolutional visual transformer (PCViT), which leverages a pyramid architecture and self-supervised pre-training to better capture multi-scale information in remote sensing images. Gong et al. [37] proposed a lightweight detection network combining Group Convolution, ShuffleNetV2, and Vision Transformer to optimize efficiency. Gao et al. [38] designed the spatio-temporal aggregation transformer (STAT) for neuromorphic datasets by incorporating density-based adaptive sampling, sparse event tensors, and lightweight triaxial vision transformers, achieving high accuracy.

While ViT-based models enhance feature representation and capture global context, they often struggle with local details and efficiency issues.

### 2.2. Detection Transformer (DETR)

Carion et al. [39] first introduced DETR, an end-to-end detector based on Transformer, which completely abandons traditional manual anchor points and the complex non-maximum suppression (NMS) component. Instead, DETR directly predicts one-to-one object sets through a binary matching mechanism. Although DETR offers several advantages, it suffers from slow convergence, high computational cost, and complicated query optimization.

To address the slow convergence, Zhu et al. [40] proposed a deformable attention module to improve the training convergence of multi-scale feature fusion. Liu et al. [41] introduced a dynamic anchor-based query formulation that uses box coordinates to enhance the similarity between queries and features, thereby alleviating the slow training issue in DETR. Li et al. [42] further proposed using real noisy bounding boxes based on DETR and Deformable DETR, which effectively reduced the difficulty of bipartite graph matching and accelerated convergence. Chen et al. [43] introduced a training method using one-to-many assignments within groups, coupled with separate decoder self-attention, to provide extra supervision and further speed up convergence. Additionally, Meng et al. [44] learned conditional space queries from decoder embeddings, allowing each cross-attention head to focus on different regions, which also contributed to faster convergence.

To tackle the high computational cost, Roh et al. [45] proposed a sparse encoder token and applied an auxiliary detection loss to it, thereby reducing computational burden while enhancing detection performance. Yao et al. [46] replaced the traditional six decoders with a single decoder, significantly boosting detection efficiency. Li et al. [47] improved the encoder’s efficiency by interleaving the update of high-level and low-level features, thereby increasing the reliability of predicted attention weights. Regarding query optimization, Chen et al. [48] represented object queries as box queries, reducing optimization difficulty while ensuring convergence speed. Wang et al. [49] introduced the concept of anchors for object queries, allowing for better query optimization. Finally, Zhao et al. [50] decoupled intra-scale interactions from cross-scale fusion to quickly process multi-scale features, improved encoding efficiency and convergence speed, and proposed queries with minimal uncertainty, thus significantly enhancing the quality of initial queries.

DETR-based approaches, though innovative in removing manual components like anchors and NMS, still face challenges in convergence speed and computational cost.

### 2.3. Loss Function for Infrared Small Target Detection

A key challenge in infrared small target detection is the severe class imbalance between the target and background pixels. To address this, researchers have developed specialized loss functions that balance localization accuracy and computational efficiency, while tackling challenges such as edge ambiguity and scale sensitivity.

In segmentation-based frameworks, precise edge delineation is critical. ISNet [51] introduces EdgeLoss, which independently computes binary cross-entropy and Dice losses for boundary regions. However, in low-contrast and noisy environments, this method may not robustly distinguish subtle edge features and can lead to misclassification. Similarly, Lv et al. [52] improved YOLOv3 for infrared targets by redesigning the edge loss function and network architecture. However, its high sensitivity to gradient changes may cause instability during training.

For bounding box-based detection, multi-component loss functions are widely adopted. Mao et al. [53] employed SoftIoU loss during training to handle ambiguous or uncertain object boundaries, leveraged Sparse SoftIoU Loss to simultaneously focus on coarse-grained and fine-grained information, and utilized Orthogonality Regularization Loss to enhance the model’s representational capacity and generalization ability. OSCAR [54] addressed class imbalance by employing Focal Loss, enhanced bounding box localization accuracy using IoU loss, and tackled the challenge of assessing small object localization quality with Quality Focal Loss. Mou et al. [55] further refined localization by applying the Complete IoU loss to multi-scale detection heads. The CIoU loss is defined as Equation (1).

(1) $$L_{\mathrm{CIoU}}=1-\mathrm{IoU}+\frac{\rho^{2}\left(b,b^{gt}\right)}{c^{2}}+\alpha v$$

where ρ is the Euclidean distance between bounding box centers, *c* is the diagonal length of the minimum enclosing box, and αv penalizes aspect ratio discrepancies, effectively addressing scale sensitivity in small target regression.

Recent advances leverage geometric distribution metrics to enhance similarity measurement. Zhu et al. [56] proposed the Normalized Wasserstein Distance (NWD) loss, which models predicted and ground-truth boxes as 2D Gaussian distributions $N(\mu_{p},\Sigma_{p})$ and $N(\mu_{gt},\Sigma_{gt})$. The NWD loss computes their Wasserstein distance as Equation (2).

(2) $$L_{\mathrm{NWD}}=\exp\left(-\frac{\sqrt{2W_{2}^{2}\left(N_{p},N_{gt}\right)}}{C}\right)$$

where $W_{2}^{2}$ is the squared Wasserstein distance and *C* is a normalization constant. This metric robustly evaluates similarity for non-overlapping or nested boxes, overcoming limitations of traditional IoU. Building on this, Zhao et al. [57] integrated NWD with Inner IoU, which focuses on the overlap between the inner regions of bounding boxes, as shown in Equation (3).

(3) $$L_{\mathrm{Inner}-\mathrm{IoU}}=1-\frac{\mathrm{Inner}-\mathrm{IoU}}{\mathrm{IoU}}$$

where Inner-IoU is computed by shrinking boxes to avoid boundary noise. The hybrid loss $L_{\mathrm{NWD}}+L_{\mathrm{Inner}-\mathrm{IoU}}$ accelerates convergence and improves precision in cluttered scenes.

But the NWD loss is sensitive to the selection of the normalization constant. It may require fine-tuning in different scenarios. Moreover, in tiny target detection, the local features of the targets are not distinct enough. Therefore, distribution-based modeling still has some limitations.

In summary, multi-component loss functions combine both coarse-grained and fine-grained information. They improve target localization accuracy to some extent. However, they also introduce multiple loss terms, which increases the complexity of parameter tuning. For extremely small targets with low contrast against the background, traditional loss functions still cannot fully capture the true shape of the target. This results in limited regression accuracy.

## 3. Materials and Methods

### 3.1. The REDETR-RISTD Model Architecture

**Figure 2:**
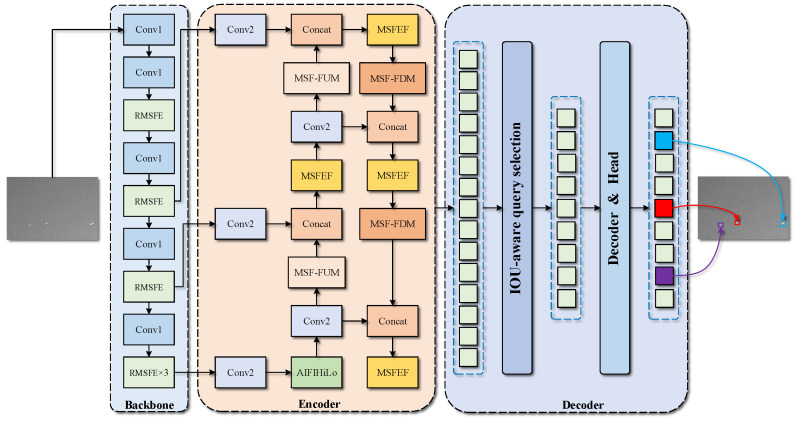
Overall structure of REDETR-RISTD. The decoder and auxiliary prediction heads iteratively refine object queries to generate category and bounding box predictions for three distinct objects, which are displayed on the right side as bounding boxes in three different colors.

The overall structure of the network is shown in Figure 2. The architecture of REDETR-RISTD consists of three key components: Backbone, Encoder, and Decoder.

In Backbone RepEMSNet, assuming that the input infrared image is $I\in R^{C\times H\times W}$, downsampling is performed using convolution with a kernel of 3 × 3 and a step size of 2 to extract fine-grained localized features in a more extensive range of the image. Reparameterized Convolution (Repconv) in the Reparameterized Multi-scale Feature Extraction (RMSFE) module makes the entire backbone network structure reparameterizable, improving the inference speed of the network without loss of accuracy. Each REMSFE can effectively and progressively increase the receptive field of the neural network by stacking the convolution kernels layer-by-layer as a 3×3 convolution while efficiently utilizing the number of parameters [58]. Feature extraction is performed through multiple branches and scales to obtain feature extraction maps with image resolutions of (H/4, W/4), (H/8, W/8), (H/16, W/16), and (H/32, W/32), respectively.

In Encoder’s Attention-based Intra-scale Feature Interaction (AIFI), we improve it by utilizing the HiLo attention mechanism [59], and the resulting AICFI module captures local details and global dependencies in the deep backbone feature map in parallel, which improves computational efficiency and detection accuracy while enhancing the robustness of the model.

A new feature fusion framework, the Multi-scale Pyramid Fusion Network (MSPFN), is designed, in which the Multi-scale Fusion and Enhancement Feature (MSFEF) module can capture and fuse the multi-scale information of the small targets in the input feature map to increase feature diversity while maintaining feature information integrity, enabling the network to learn better and understand the small target features. The MSFEF module captures and fuses the multi-scale information of small targets in the input feature map to increase feature diversity while maintaining the integrity of the feature information, which enables the network to learn better and understand the small target features. The Multi-scale Feature Fusion Downsampling Module (MSF-FDM) retains the multi-scale information of infrared small target features while reducing the size of the feature map. It focuses on extracting local features of small targets and can effectively capture global contextual information. The Multi-scale Feature Fusion Upsampling Module (MSF-FUM) enhances the representation of feature maps by multipath feature extraction and fusion during upsampling, enabling efficient upsampling while maintaining detailed and global information. Finally, a new loss function, EnIoU loss, is designed to accelerate the convergence of network training.

### 3.2. Improved Feature Extraction Network RepEMSNet

**Figure 3:**
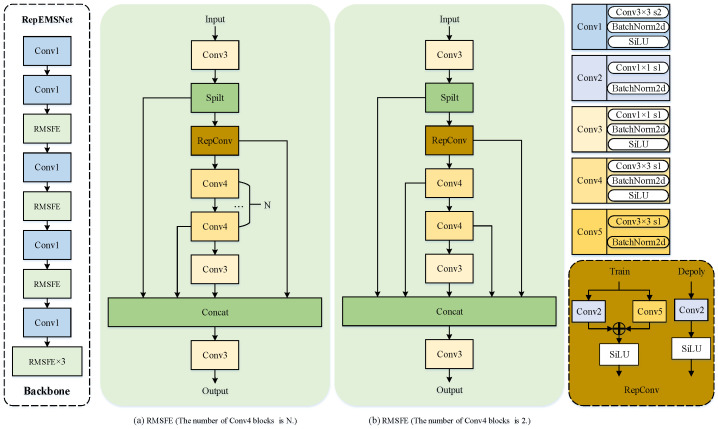
Structure of RepEMSNet. The overall architecture of the network is illustrated, with the right side detailing the structural composition of Conv1 through Conv5 layers and the RepConv module.

Figure 3 clearly shows the architecture design of the proposed backbone network, RepEMSNet. The network input is defined as a 3D tensor $X_{0}\in R^{H_{0}\times W_{0}\times 3}$, where $H_{0},\;W_{0}$ represents the spatial resolution of the infrared image. First, to rapidly compress redundant spatial data to effectively suppress interference from large homogeneous background regions in infrared images, while selectively extracting low-frequency background information encompassing scene geometric contours and temperature gradient distribution patterns, the input infrared image tensor $X_{0}\in R^{H_{0}\times W_{0}\times 3}$ undergoes initial feature extraction and downsampling via a convolutional module parameterized as (3,64;3,2), as shown in Equation (4).

(4) $$P_{1}=C\left(3,64;3,2\right)\left(X_{0}\right)$$

where $C(c_{\mathrm{in}},c_{\mathrm{out}};k,s)$ denotes the standard convolutional operation in Equation (5).

(5) $$C\left(x\right)=\sigma (\mathrm{BN}\left(\mathrm{Conv}2D\left(c_{\mathrm{in}},c_{\mathrm{out}},k,s\right)\left(x\right)\right))$$

where σ denotes the SiLU activation function, Conv2D represents the standard 2D convolution operation, and BN stands for batch normalization (BN). The symbols $c_{\mathrm{in}},c_{\mathrm{out}},k,s$ represent the input channels, output channels, kernel size, and stride of the convolutional layer, respectively. In the function, *x* is the independent variable and does not represent any specific numerical value. All subsequent occurrences of *x* retain this definition. This operation reduces the resolution of $X_{0}$ to $H_{0}/2\times W_{0}/2$ with 64 output channels.

Next, to enhance feature diversity for addressing the ambiguity of infrared small targets caused by low SNR, while emphasizing critical characteristics such as target edge sharpness and local contrast enhancement, further downsampling is performed using a convolutional layer parameterized as (64,128;3,2), as shown in Equation (6). By implementing spatial compression, it reduces computational complexity in subsequent stages while preserving the positional information of targets.

(6) $$P_{2}=C\left(64,128;3,2\right)\left(P_{1}\right)$$

where the output feature map $P_{2}\in R^{\frac{H_{0}}{4}\times \frac{W_{0}}{4}}$ has 128 channels.

To further enhance critical target characteristics while maintaining real-time processing capabilities and suppressing interference from complex backgrounds, the features $P_{2}$ are directed into the RMSFE module for enhancement through the following steps in Equations (7) to (12).

(1) Channel Expansion and Splitting. The input $P_{2}$ undergoes 1×1 convolution parameterized as (128,128;1,1) in Equation (7).

(7) $$F=C\left(128,128;1,1\right)\left(P_{2}\right)$$

*F* is then split into two components along the channel dimension $F^{\left(a\right)},F^{\left(b\right)}$ in Equation (8).

(8) $$\left\{F^{\left(a\right)},F^{\left(b\right)}\right\}=\mathrm{split}\left(F,1\right)$$

where $F^{\left(a\right)},F^{\left(b\right)}\in R^{\frac{H_{0}}{4}\times \frac{W_{0}}{4}\times 64}$. $F^{\left(a\right)}$ preserves the integrity of original channel-wise data to prevent small targets from being diluted or lost during complex nonlinear transformations, while $F^{\left(b\right)}$ serves as an enhancement branch that employs targeted feature amplification mechanisms to significantly improve both the SNR and spatial activation intensity in target regions.

(2) Residual Feature Reconstruction. The branch $F^{\left(b\right)}$ undergoes processing in Equation (9) via the RepConv module defined in Equation (10). To ensure real-time processing capabilities, the RepConv leverages multi-scale convolutional kernel fusion to amplify edge sharpness and local contrast in target regions while suppressing background clutter.

(9) $$G_{0}=R\left(64,32;3,1\right)\left(F^{\left(b\right)}\right)$$

where R(·) during training adopts a multi-branch structure in Equation (10).

(10) $$R\left(x\right)=\sigma \left(C(64,32;3,1)(x)+C(64,32;1,1)(x)+\mathrm{BN}(x)\right)$$

(3) Recursive Convolutional Chain. The feature maps undergo progressive refinement through n−1 layers of recursive convolutional operations as defined in Equation (11), which iteratively enhances multi-scale feature to amplify sub-pixel details and strengthen the discriminative capability of microscopic targets.

(11) $$G_{k}=C\left(32,32;3,1\right)\left(G_{k-1}\right),\;k\in \left\{1,\ldots ,n-1\right\}$$

(4) Multi-Level Feature Fusion. All hierarchical representations are concatenated and dimensionally compressed through the 1×1 convolutional operation defined in Equation (12). This architecture synergistically enhances cross-scale feature complementarity, strengthens background clutter suppression capability, and preserves sub-pixel positional accuracy by maintaining native spatial resolution, thus effectively mitigating localization drift caused by conventional downsampling.

(12) $$P_{3}=C\left(d_{\mathrm{total}},128;1,1\right)\left(\mathrm{concat}(F^{\left(a\right)},\left\{G_{k}\right\},G_{\mathrm{fin}})\right)$$

where $d_{\mathrm{total}}=64+32\left(n+1\right)$ and $G_{\mathrm{fin}}=C\left(32,32;1,1\right)\left(Gn-1\right)$. The output resolution remains $\frac{H_{0}}{4}\times \frac{W_{0}}{4}\times 128$.

The preceding sections detail the computational processes of the first three core modules in the RepEMSNet backbone architecture. While subsequent network stages follow analogous feature transformation paradigms, we focus here on enumerating the critical parameters of RepEMSNet as summarized in Table 1.

Notably, the scaling factor *s* within the RMSFE module regulates the receptive fields and feature diversity through dynamic adjustment of intermediate channel dimensions. Specifically, s=1 amplifies high-frequency feature extraction capability, enhancing sensitivity to infrared small targets by expanding intermediate channels to 384. s=0.5 optimizes background suppression, reducing the false alarm rate (FAR) through channel compression. The multi-scale output feature maps generated by the backbone network RepEMSNet are hierarchically denoted as S1, S2, S3, S4, and S5, corresponding to spatial–semantic information at progressively increasing receptive fields.

### 3.3. Improved Feature Interaction AICFI

**Figure 4:**
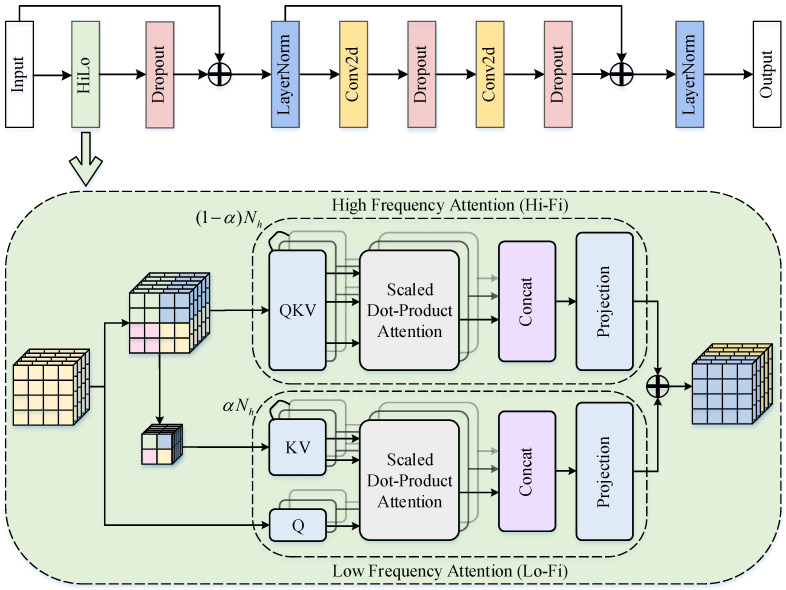
Framework of AICFI. The main architecture of AICFI is presented in the upper portion of the figure, while the lower portion illustrates the internal structure of the HiLo attention mechanism.

Existing studies [50] have demonstrated that applying self-attention mechanisms to high-level semantic-rich features can effectively model conceptual entity relationships, thereby enhancing object localization and recognition capabilities in downstream modules. However, conventional self-attention frameworks face critical limitations in infrared small target detection: (1) Standard attention mechanisms struggle to distinguish between high-frequency edge noise and low-frequency background radiation due to their indiscriminate spectral treatment. (2) Fixed-window local attention mechanisms lack adaptability to dynamic target scale variations. (3) The implementation of the global self-attention mechanism requires more computational resources. To overcome these limitations, we present the Attention-guided Intra-scale Contextual Feature Interaction (AICFI) module, a novel hierarchical architecture specifically designed for infrared small target characterization. As illustrated in Figure 4, the AICFI module employs a systematic frequency-aware decomposition-to-fusion paradigm. This innovative architecture employs a frequency-decoupling strategy combined with a dynamic fusion mechanism, implementing a hierarchical attention framework to enhance discriminative feature representation for infrared small targets.

As shown in Figure 4, $N_{h}$ denotes the total number of self-attentive heads in the layer and the ratio of the division between high-frequency and low-frequency heads. α serves as a balancing factor that dynamically adjusts the allocation ratio of attention heads between the high-frequency and low-frequency branches.

First, the input feature map $X_{1}\in R^{c_{1\mathrm{in}}\times H_{1}\times W_{1}}$ is implicitly mapped to a dual-path interaction space through a parameterized feature decomposition strategy, where the high-frequency path (Hi-Fi) and low-frequency path (Lo-Fi) are dynamically allocated computational resources by the hyperparameters α∈[0,1] and the total number of attention heads $N_{h}$. The high-frequency path is allocated $N_{h}\left(1-\alpha \right)$ heads to focus on fine-grained interactions within local windows, while the low-frequency path is allocated $N_{h}\alpha$ heads to handle sparse global semantic modeling.

Second, the high-frequency path divides the input feature into $\frac{H_{2}}{s}\times \frac{W_{2}}{s}$ local windows (where *s* is the window size). Within each window, pixel-level dependency relationships are modeled via multi-head self-attention (MHSA), as shown in Equation (13).

(13) $$X_{\mathrm{Hi}-\mathrm{Fi}}=\overset{N_{h}\left(1-\alpha \right)}{\underset{i=1}{⨁}}\mathrm{Softmax}\left(\frac{Q_{i}K_{i}^{T}}{\sqrt{d}}\right)V_{i}$$

where ⨁ denotes the channel-wise concatenation of multi-head outputs, and *d* represents the dimension per attention head. This operation reinforces high-order statistical quantities such as target edges and textures within constrained receptive fields while suppressing high-frequency noise.

Simultaneously, the low-frequency path compresses the feature resolution to $\frac{H_{2}}{s}\times \frac{W_{2}}{s}$ through spatial downsampling and performs global multi-head attention in the compressed space, as shown in Equation (14).

(14) $$X_{\mathrm{Lo}-\mathrm{Fi}}={\mathrm{UP}}_{s}\left(\overset{N_{h}\alpha }{\underset{j=1}{⨁}}\mathrm{Softmax}\left(\frac{{\tilde{Q}}_{j}{\tilde{K}}_{j}^{T}}{\sqrt{d}}\right){\tilde{V}}_{j}\right)$$

where $\mathrm{UP}s\left(\cdot \right)$ denotes bilinear interpolation upsampling. The low-frequency path enhances semantic discriminability between targets and backgrounds by leveraging sparsified features to filter out local perturbations.

Next, the dual-path features undergo multi-granularity fusion through channel-wise projection and concatenation, as shown in Equation (15).

(15) $$X_{1\mathrm{fused}}=P_{h}\left(X_{\mathrm{Hi}-\mathrm{Fi}}\right)⊕P_{l}\left(X_{\mathrm{Lo}-\mathrm{Fi}}\right)$$

Here, $P_{h}\left(\cdot \right)$ and $P_{l}\left(\cdot \right)$ are learnable channel projection matrices, and ⊕ represents the concatenation operation. The fused feature retains high-frequency details and low-frequency semantics, forming a complementary representation.

Finally, gradient propagation is stabilized via residual connections and a pre-normalization strategy, yielding the final output $X_{\mathrm{out}}$ in Equation (16) of AICFI.

(16) $$X_{\mathrm{out}}=\mathrm{LayerNorm}\left(X_{\mathrm{in}}+W_{2}\cdot \mathrm{GELU}\left(W_{1}\cdot X_{\mathrm{fused}}\right)\right)$$

where $W1\in R^{c_{1\mathrm{in}}\times 4c_{1\mathrm{in}}}$ and $W2\in R^{4c_{1\mathrm{in}}\times c_{1\mathrm{in}}}$ are the weight matrices of the multi-layer perceptron (MLP). This process ensures stable optimization in deep networks, producing the enhanced output feature $X_{\mathrm{out}}\in R^{c_{1\mathrm{in}}\times H_{1}\times W_{1}}$.

### 3.4. Feature Fusion Framework MSPFN

**Figure 5:**
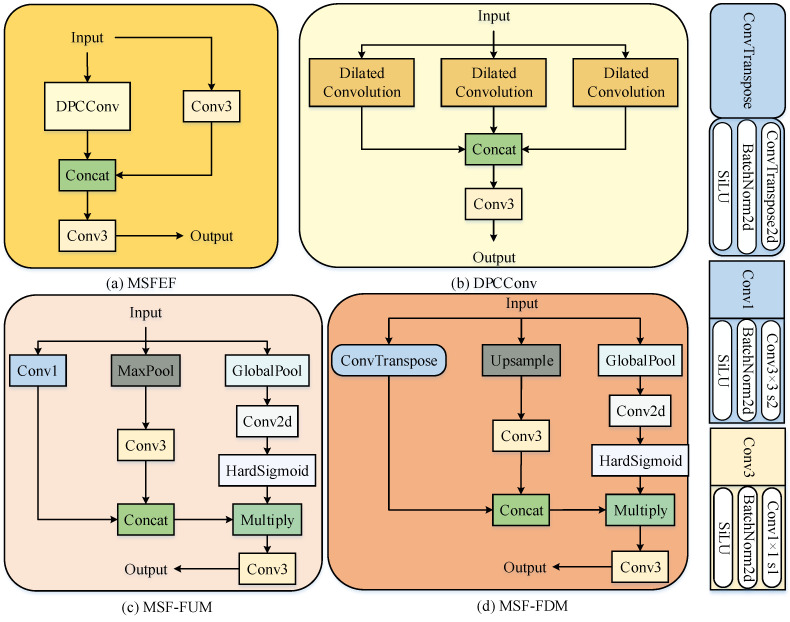
Overall structure of MSPFN. The figure illustrates the main components (**a**) MSFEF, (**b**) DPCConv, (**c**) MSF-FUM, and (**d**) MSF-FDM, along with the internal structures of the ConvTranspose, Conv1, and Conv3 modules shown on the right.

In order to solve the problems of detail loss in multi-scale feature fusion, insufficient ability to model cross-scale context, and semantic differences between features at different levels of the feature pyramid in traditional feature fusion methods, we propose a novel feature fusion framework, MSPFN, which combines the pyramid feature fusion principle and incorporates the MSFEF module, MSF-FDM downsampling, and MSF-FUM upsampling. The overall structure of MSPFN is shown in Figure 5.

#### 3.4.1. Multi-Scale Feature Fusion Upsampling Module

The structure of MSF-FUM is shown in Figure 5c. It achieves refined reconstruction of high-resolution features through a sequential process. First, the input feature map $X_{\mathrm{iu}}\in R^{c_{\mathrm{iu}}\times H_{\mathrm{iu}}\times W_{\mathrm{iu}}}$ undergoes Global Average Pooling (GAP) to compress spatial dimensions, generating channel-wise global statistics $X_{\mathrm{pool}}\in R^{c_{\mathrm{iu}}\times 1\times 1}$. This step captures global semantic information for each channel to guide subsequent channel gating. Second, the global statistics pass through a channel gating module composed of a 1×1 convolution and a Hardsigmoid activation function, producing a dynamic weight matrix $G_{\mathrm{iu}}\in {\left[0,1\right]}^{c_{\mathrm{iu}}\times 1\times 1}$ as shown in Equation (17).

(17) $$G_{\mathrm{iu}}=\sigma \left(C\left(c_{\mathrm{iu}},c_{\mathrm{iu}};1\right)\left(\mathrm{GAP}\left(X_{\mathrm{iu}}\right)\right)\right)$$

where σ denotes the Hardsigmoid activation function. This matrix adaptively regulates channel contributions during feature fusion, suppressing noise and enhancing critical features. Next, the module processes the input through two parallel upsampling branches. Branch 1 uses a transposed convolution with learnable kernels to restore spatial resolution. The output $F_{\mathrm{up}1}$ is derived by Equation (18).

(18) $$F_{\mathrm{up}1}=T\left(c_{\mathrm{iu}},c_{\mathrm{iu}}/2;2,2\right)\left(X_{\mathrm{iu}}\right)$$

where $T(c_{\mathrm{in}},c_{\mathrm{out}};k,s)$ is defined in Equation (19).

(19) $$T\left(c_{\mathrm{in}},c_{\mathrm{out}};k,s\right)=\sigma (\mathrm{BN}\left(\mathrm{ConvTranspose}2d\left(k,s\right)\right)$$

Here, σ represents the SiLU activation function. This operation restores the spatial resolution to $2H_{\mathrm{iu}}\times 2W_{\mathrm{iu}}$ while enhancing nonlinearity via BN and SiLU.

Branch 2 combines bilinear interpolation and lightweight convolution. First, the feature map is upsampled to $2H_{\mathrm{iu}}\times 2W_{\mathrm{iu}}$ via bilinear interpolation I(·), followed by a 1×1 standard convolutional operation to adjust channels as shown in Equation (20).

(20) $$F_{\mathrm{up}2}=C\left(c_{\mathrm{iu}},c_{\mathrm{iu}}/2;1,1\right)\left(I\left(X_{\mathrm{iu}}\right)\right)$$

where I(·) denotes the bilinear interpolation operator. These two branches achieve multi-scale modeling of detailed information from the perspectives of local structure learning and global topology, respectively, forming complementary feature representations. The outputs of both branches are concatenated along the channel dimension to form an intermediate feature $F_{\mathrm{ucat}}=F_{\mathrm{upl}}⊕F_{\mathrm{up}2}\in R^{c_{\mathrm{in}}\times 2H_{\mathrm{iu}}\times 2W_{\mathrm{iu}}}$. The channel gating weights $G_{\mathrm{iu}}$ are broadcast to match the spatial dimensions of $F_{\mathrm{ucat}}$ and applied via channel-wise multiplication as shown in Equation (21).

(21) $$F_{\mathrm{uweighted}}=G_{\mathrm{iu}}⊙F_{\mathrm{ucat}}$$

This operation dynamically enhances target-related channels and suppresses background noise based on global semantic cues. Finally, the weighted features undergo a standard convolutional operation for channel fusion and nonlinear transformation, producing the high-resolution output $F_{\mathrm{uout}}$ in Equation (22).

(22) $$F_{\mathrm{uout}}=C\left(c_{\mathrm{iu}},c_{\mathrm{iu}};1\right)\left(F_{\mathrm{uweighted}}\right)$$

#### 3.4.2. Multi-Scale Feature Fusion Downsampling Module

The structure of MSF-FDM is shown in Figure 5d. It reduces the spatial resolution of feature maps while retaining key semantic information and suppressing background noise through multi-branch downsampling strategies and adaptive channel gating mechanisms. First, the input feature map $X_{\mathrm{id}}\in R^{c_{\mathrm{id}}\times H_{\mathrm{id}}\times W_{\mathrm{id}}}$ undergoes GAP to compress spatial dimensions, generating channel-wise global statistics $X_{\mathrm{pool}}\in R^{c_{\mathrm{id}}\times 1\times 1}$. This step captures global semantic information for each channel, providing prior guidance for channel gating.

Second, the global statistics pass through a channel gating module composed of a 1×1 convolution and a Hardsigmoid activation function, producing a dynamic weight matrix $G_{\mathrm{id}}\in {\left[0,1\right]}^{c_{\mathrm{id}}\times 1\times 1}$ as shown in Equation (23).

(23) $$G_{\mathrm{id}}=\sigma \left(C\left(c_{\mathrm{id}},c_{\mathrm{id}};1\right)\left(\mathrm{GAP}\left(X_{\mathrm{id}}\right)\right)\right)$$

where σ denotes the Hardsigmoid function. This matrix $G_{\mathrm{id}}$ adaptively regulates the importance of channels during downsampling, suppressing redundant features and enhancing target-related contextual information. Next, the module processes the input through two parallel downsampling branches. Branch 1 uses a standard convolution operation with a 3×3 kernel and stride 2 for spatial downsampling in Equation (24).

(24) $$F_{\mathrm{down}1}=C\left(c_{\mathrm{id}},c_{\mathrm{id}}/2;3,2\right)\left(X_{\mathrm{id}}\right)$$

The output feature map $F_{\mathrm{down}1}$ has dimensions of H/2×W/2. This branch captures multi-scale context by expanding the local receptive field while reducing resolution. Branch 2 combines MaxPooling and lightweight convolution. First, a 2×2 MaxPool operation reduces the spatial size to H/2×W/2, followed by a 1×1 convolution for channel adjustment to obtain $F_{\mathrm{down}2}$ in Equation (25).

(25) $$F_{\mathrm{down}2}=C\left(c_{\mathrm{id}},c_{\mathrm{id}/2};1\right)\left(\mathrm{MaxPool}\left(X_{\mathrm{id}}\right)\right)$$

where MaxPool(·) denotes the max pooling operation. This branch preserves salient local structures, achieving channel compression and nonlinear transformation via a standard convolution operation with a 1×1 kernel and stride 1. Then, the outputs of both branches are concatenated along the channel dimension to form an intermediate feature $F_{\mathrm{dcat}}=F_{\mathrm{down}1}⊕F_{\mathrm{down}2}$, where $F_{\mathrm{dcat}}\in R^{c_{\mathrm{id}}\times H_{\mathrm{id}}/2\times W_{\mathrm{id}}/2}$. The channel gating weights $G_{\mathrm{id}}$ are broadcast to match the spatial dimensions of $F_{\mathrm{dcat}}$ and applied via channel-wise multiplication as shown in Equation (26).

(26) $$F_{\mathrm{dweighted}}=G_{\mathrm{id}}⊙F_{\mathrm{dcat}}$$

This operation adaptively enhances target-related channel responses and suppresses noise based on global semantics. Finally, the weighted features undergo a standard convolution for channel fusion and nonlinear transformation, producing the low-resolution output feature map $F_{\mathrm{dout}}$ in Equation (27).

(27) $$F_{\mathrm{dout}}=C\left(c_{\mathrm{dout}},c_{\mathrm{dout}};1\right)\left(\left(F_{\mathrm{dweighted}}\right)\right)$$

#### 3.4.3. Multi-Scale Feature Fusion Enhancement Module

The structure of MSFEF is illustrated in Figure 5a,b. MSFEF addresses the challenges of varying target scales and complex backgrounds in infrared small target detection through Cross Stage Partial connections and multi-scale parallel atrous convolutions, enabling multi-granularity semantic modeling and efficient fusion of input features.

First, the input feature map $X_{2}\in R^{c_{2\mathrm{in}}\times H_{2}\times W_{2}}$ undergoes channel compression via two independent 1×1 standard convolution operations, generating the main branch feature $X_{21}$ and residual branch feature $X_{22}$ as shown in Equations (28) and (29).

(28) $$X_{21}=C\left(c_{2}\mathrm{in},c_{\mathrm{hid}};1\right)\left(X_{2}\right)$$

(29) $$X_{22}=C\left(c_{2}\mathrm{in},c_{\mathrm{hid}};1\right)\left(X_{2}\right)$$

where $c_{\mathrm{hid}}=\left[c_{2\mathrm{out}}\cdot e\right]$ denotes the hidden layer channels, with e=0.5 as the default setting.

Second, the main branch feature $X_{21}$ is fed into the DPCConv module, where parallel atrous convolutions with different dilation rates *d* extract multi-scale features $Y_{1}$,$Y_{2}$, and $Y_{3}$ in Equations (30) to (32).

(30) $$Y_{1}=C\left(c_{\mathrm{hid}},c_{\mathrm{hid}};3,d=1\right)\left(X_{21}\right)$$

(31) $$Y_{2}=C\left(c_{\mathrm{hid}},c_{\mathrm{hid}}/2;3,d=2\right)\left(X_{21}\right)$$

(32) $$Y_{3}=C\left(c_{\mathrm{hid}},c_{\mathrm{hid}}/2;3,d=3\right)\left(X_{21}\right)$$

where dilated convolution with d=1 mainly focuses on the local details of infrared small targets. Dilated convolution with d=2 primarily models the local context of the image. And dilated convolution with d=3 is mainly used to suppress background noise. Next, $Y_{1}$, $Y_{2}$, and $Y_{3}$ are concatenated along the channel dimension to form $Y_{\mathrm{cat}}=Y_{1}⊕Y_{2}⊕Y_{3}\in R^{2c_{\mathrm{hid}}\times H_{2}\times W_{2}}$. A 1×1 standard convolution operation then compresses channels and fuses multi-scale information in Equation (33).

(33) $$Y_{\mathrm{fused}}=C\left(2c_{\mathrm{hid}},c_{\mathrm{hid}};1\right)\left(Y_{\mathrm{cat}}\right)$$

This step enhances sensitivity to small target features while suppressing noise by jointly modeling local details and global context. Subsequently, the multi-scale fused feature $Y_{\mathrm{fused}}$ is concatenated with the residual branch feature $X_{22}$ in Equation (34).

(34) $$Z_{\mathrm{cat}}=Y_{\mathrm{fused}}⊕X_{22}\in R^{2c_{\mathrm{hid}}\times H_{2}\times W_{2}}$$

The residual branch preserves low-frequency components to mitigate the signal attenuation caused by multi-scale convolutions. Finally, a 1×1 standard convolution adjusts the channels of $Z_{\mathrm{cat}}$ to generate the output feature $F_{\mathrm{mout}}$ in Equation (35).

(35) $$F_{\mathrm{mout}}=C\left(2c_{\mathrm{hid}},c_{\mathrm{mout}};1\right)\left(Z_{\mathrm{cat}}\right)\in R^{c_{\mathrm{mout}}\times H_{2}\times W_{2}}$$

This output integrates multi-scale semantics while preserving critical target details, ensuring robust detection in complex infrared scenes.

### 3.5. Loss Function EnIoU Loss

**Figure 6:**
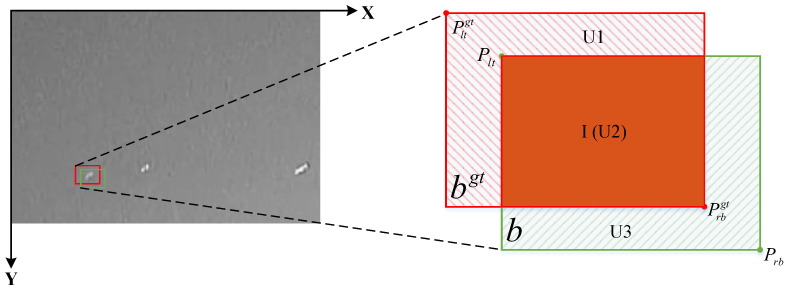
Diagram illustrating the loss function. The red box represents the ground-truth box, while the green box represents the predicted bounding box.

The current IoU-based bounding box regression loss (BBRL) mainly accelerates convergence by adding new Loss terms while ignoring the shortcomings of the IoU Loss itself. Since the samples need to be differentiated in the regression process, using auxiliary bounding boxes of different scales to calculate the loss can effectively accelerate the regression process of the bounding box. Therefore, in the model training strategy, we use a smaller auxiliary bounding box to compute the loss for regressions with high-IoU samples and vice versa for low-IoU samples. To address the problem that the Generalized Intersection over Union (GIoU) [60] loss in the traditional bounding box regression loss function in RT-DETR is sensitive to the scale of small targets, we not only take into account the degree of overlap between the prediction bounding box and the ground-truth (GT) box but also the positional accuracy, which makes the prediction bounding box closer to the GT box. Equations (36) to (40) represent the construction process of EnIoU loss.

(36) $$I=\left(\min\left(b_{r}^{gt},b_{r}\right)-\max\left(b_{l}^{gt},b_{l}\right)\right)\times \left(\min\left(b_{b}^{gt},b_{b}\right)-\max\left(b_{t}^{gt},b_{t}\right)\right)$$

(37) $$U=\left(\frac{w^{gt}\times h^{gt}}{ratio^{2}}\right)+\left(\frac{w\times h}{ratio^{2}}\right)-I+\varepsilon =U_{1}+U_{2}+U_{3}$$

(38) $$D_{1}={∥P_{lt}-P_{lt}^{gt}∥}_{2}$$

(39) $$D_{2}={∥P_{rb}-P_{rb}^{gt}∥}_{2}$$

(40) $$L_{\mathrm{EnIoU}}=1-\frac{\;I}{\;U}+\frac{D_{1}}{2}+\frac{D_{2}}{2}$$

where $b^{gt}$ and *b* denote the GT box and anchor box, respectively, as shown in the red bounding box and green bounding box in Figure 6. $\min\left(b_{r}^{gt},b_{r}\right)$ denotes the right border of the area where the two bounding boxes overlap. $\max\left(b_{l}^{gt},b_{l}\right)$ denotes the left border of the area where the two bounding boxes overlap. $\min\left(b_{b}^{gt},b_{b}\right)$ indicates the lower boundary of the area where the two bounding boxes overlap. $\max\left(b_{t}^{gt},b_{t}\right)$ denotes the upper boundary of the area where the two bounding boxes overlap. *I* is the area of the portion of the intersection of the two bounding boxes, as shown in the dark filled area of Figure 6. $w^{gt}$ and $h^{gt}$ denote the width and height of the GT box, while the width and height of the prediction bounding box are denoted as *w* and *h*, respectively. The ratio is the ratio factor that controls the size of the auxiliary box. ε is a regular term. *U* is the total area occupied by the two bounding boxes, $U_{1}$, $U_{2}$, and $U_{3}$ denote the three components of *U*, and there is $I=U_{2}$, as shown in Figure 6. $P_{lt}=\left(x_{lt},y_{lt}\right)$, $P_{lt}^{gt}=\left(x_{lt}^{gt},y_{lt}^{gt}\right)$, $P_{lb}=\left(x_{lb},y_{lb}\right)$, $P_{rb}^{gt}=\left(x_{rb}^{gt},y_{rb}^{gt}\right)$ denotes the top-left and top-right vertices of the predicted and true boxes in Figure 6, respectively. ${∥\cdot ∥}_{2}$ denotes the Euclidean distance.

## 4. Results

### 4.1. Dataset

In this study, we use three open datasets to evaluate the performance of the proposed detector: SIRSTv2 [54], IRSTD-1k [51], and NUDT-SIRST [19]. To ensure a proper evaluation, we divide each dataset into training, validation, and test sets in a ratio of 7:1:2, and the final results are obtained from the test set. Next, we will provide a detailed introduction to the selected datasets.

The SIRSTv2 dataset, proposed by Dai Yimian from Nanjing University of Aeronautics and Astronautics, is a publicly available single-frame infrared small object detection dataset. This dataset contains 1024 typical infrared images, most of which have a resolution of 1280 × 1024, making it one of the highest-resolution datasets available for infrared small target detection. The images in SIRSTv2 are extracted from real-world video sequences, capturing various complex scenarios. These include urban environments where background interference, such as cranes and streetlights, resembles the appearance of targets, making detection more challenging. The dataset reflects the difficulties of infrared small object detection in realistic settings, where most targets are dim and difficult to distinguish from cluttered backgrounds due to low contrast and high noise. Unlike traditional approaches that rely on target saliency or low-rank sparse decomposition, SIRSTv2 requires a more advanced method that can leverage high-level semantic understanding of the entire image to effectively distinguish targets from non-target interference. The distribution of objects in the images is random, and many of the targets are not easily separable from the background, requiring robust contextual information for accurate detection.

The IRSTD-1k dataset, proposed by Zhang Mingjin and colleagues from Xidian University, is a publicly available benchmark dataset for infrared small object detection. The dataset covers a broader range of extreme conditions and challenging environments by integrating multi-source infrared imaging equipment and dynamic acquisitions from real-world scenarios. IRSTD-1k contains 1000 infrared images with a resolution of 512 × 512, annotated with 1520 infrared small objects. The data are sourced from both static and dynamic acquisitions across four major environmental categories: sky backgrounds (static cloud layers, dynamic flying birds), urban backgrounds (building thermal radiation, vehicle exhaust), natural backgrounds (forests, deserts), and extreme weather conditions (haze, rain, snow). The imaging spectrum spans short-wave, mid-wave, and long-wave infrared bands to simulate the imaging characteristics of different sensors. To enhance data diversity, synthetic noise (Gaussian noise, Poisson noise) is added to a subset of images, reducing the SNR. Approximately 40% of the images exhibit an SNR below 2 dB, where background noise nearly overwhelms object signals. The dataset highlights multi-scale object characteristics: approximately 30% of objects are smaller than 3 × 3 pixels (occupying less than 0.01% of the 512 × 512 image area), with some objects being single-pixel points (pixel coverage: 0.02%). The minimum object size is 1 × 1 pixels, while the maximum reaches 15 × 15 pixels. Regarding intensity distribution, only 20% of objects are the brightest regions globally, whereas 80% exhibit grayscale values similar to or lower than the background. The grayscale difference between objects and backgrounds is often below 10 in haze and desert scenarios.

The NUDT-SIRST dataset, developed by researchers from the National University of Defense Technology, is a publicly available synthesized single-frame infrared small target dataset.

Unlike traditional datasets built from object motion sequences, NUDT-SIRST is generated using simulation techniques that ensure highly accurate pixel-level annotations while offering extensive control over target characteristics and background complexity. The dataset comprises 1327 infrared images of 256 × 256 resolution, encompassing a diverse range of target categories and sizes embedded in richly cluttered and noisy backgrounds. Similar to real infrared scenarios, the images contain significant noise and low SNR, with the small targets often occupying less than 0.02% of the total image area—that is, in a standard 256 × 256 image, the target pixels typically cover an area smaller than 4 × 4 pixels. Furthermore, only about 35% of the objects appear as the brightest regions in the image, while the remaining 65% exhibit grayscale values similar to or even lower than their surroundings. This characteristic renders simple saliency-based approaches ineffective and necessitates the use of both global and local contextual information for reliable detection.

### 4.2. Evaluation Metrics

The three datasets contain only one infrared small target class. Therefore, we use AP50, AP50-95, and recall as precision evaluation metrics; computational and parametric quantities to measure the model’s time complexity and space complexity; and the precision–recall (PR) curve to verify the performance of the algorithm.

Wherein AP denotes average precision, AP50 denotes the average precision when the IoU of the detection model is set to the average precision of 0.5, and AP50-95 denotes the average value of the precision obtained when the IoU of the detection model is selected in the range of 0.5 to 0.95, and the value of AP can be obtained from the area under the PR curve. The unit of parametric quantity is M. Usually, the smaller the model computation and parametric quantity, the higher the FPS of the detection network, though this is not always the case.

Before describing the PR curve, it is necessary to clarify that the infrared small target detection results fall under two categories—target and non-target; therefore, this type of detection problem can be seen as a binary classification problem. Binary classification problems include four kinds of results: TP (true positive, both predicted and actual positive samples), FP (false positive, predicted positive samples, actual negative samples), FN (false positive, predicted negative samples, actual positive samples), and TN (true negative, both predicted and actual negative samples). The horizontal and vertical coordinates of the PR curves are recall (R) and precision (P), respectively. R denotes the proportion of all positive samples that are correctly predicted to be positive samples, and P denotes the proportion of samples predicted to be positive samples that are positive samples. R, P, and AP can be computed by Equations (41) to (43).

(41) $$R=\frac{TP}{TP+FN}$$

(42) $$P=\frac{TP}{TP+FP}$$

(43) $$AP=\int_{0}^{1}P(R)d(R)$$

We also used target-level evaluation indicators, the probability of detection ($P_{d}$) and false alarm rate ($F_{a}$), to comprehensively evaluate the detector’s performance from multiple aspects.

Formally, the probability of detection is obtained by Equation (44).

(44) $$P_{d}=\frac{L_{tp}}{L_{all}}$$

where $L_{tp}$ is the number of correctly predicted targets and $L_{all}$ is the number of all targets. The probability of a false alarm rate is obtained by Equation (45).

(45) $$F_{a}=\frac{P_{f}}{P_{all}}$$

where $P_{f}$ is the number of targets for the error prediction, and $P_{all}$ is the target quantity of all predictions.

### 4.3. Training Details

The hardware configuration for the experiment is shown in Table 2.

The method proposed in this paper is implemented using Pytorch. The training parameters are set as follows: the image size is uniformly adjusted to 640 × 640. The YOLO series network selects the SGD optimizer with high computational efficiency and fast convergence speed for network training. The RTDETR series uses the AdamW optimizer. The momentum is set to 0.937. The initial learning rate is 0.01. The decay factor is 0.0001, and the training epoch is 500. A learning rate preheating strategy is used to improve the stability and performance of model training and reduce the risk of oscillations and gradient explosion at the beginning of training. The warmup epoch is set to 3, the warmup momentum is set to 0.8, and the learning rate of the warmup bias parameter is set to 0.1.

The boundary box-based object detection method TOOD uses the SGD optimizer with an initial learning rate of 0.0001, final learning rate of 0.0001, decay factor of 0.0001, and momentum of 0.9. Sparse R-CNN uses the AdamW optimizer with an initial learning rate of 0.000025, final learning rate of 0.0001, and decay factor of 0.0001. Mask R-CNN uses the SGD optimizer with an initial learning rate of 0.0002, final learning rate of 0.0001, decay factor of 0.0001, and momentum of 0.9. DINO uses the AdamW optimizer with an initial learning rate of 0.0001, final learning rate of 0.0001, and decay factor of 0.0001. All four methods use the MultiStepLR learning rate scheduler.

The segmentation-based detection methods ACM, ALCNet, DNANet, ISTDU-Net, RDIAN, and OSCAR use the default parameters from the original code. ACM, ALCNet, ISTDU-Net, and RDIAN use the Adam optimizer with a learning rate of 0.0005, and the learning rate scheduler is MultiStepLR for all of them. DNANet uses the Adagrad optimizer with a learning rate of 0.05 and a learning rate scheduler of CosineAnnealingLR for training. OSCAR uses the AdamW optimizer with a learning rate of 0.002 and a decay factor of 0.05.

### 4.4. Results and Analysis

#### 4.4.1. Quantitative Analysis

The experimental results are shown in Table 3. REDETR-RISTD is compared with advanced target detectors. These include the YOLO series, DETR series, R-CNN series, and TOOD. On the SIRSTv2 dataset, REDETR-RISTD achieves a precision of 0.991, a recall of 0.927, an AP50 of 0.965, and an AP50-95 of 0.516. On the IRSTD-1k dataset, it obtains a precision of 0.991, a recall of 0.979, an AP50 of 0.985, and an AP50-95 of 0.745. REDETR-RISTD outperforms other models on these two datasets.

On the NUDT-SIRST dataset, REDETR-RISTD ranks second in detection metrics. Its recall is 0.979, its AP50 is 0.985, and its AP50-95 is 0.786. It is lower than the RT-DETR-HGNetv2-L model by 0.7% in recall, 1% in AP50, and 2.3% in AP50-95. The model size of REDETR-RISTD is reduced by 56.8% compared to RT-DETR-HGNetv2-L.

These results show that REDETR-RISTD performs very well on small targets. It is designed for detecting small objects. On the NUDT-SIRST dataset, the targets are relatively large. The algorithm does not fully realize its potential in this case, but it still shows strong detection capability. The high performance and lower model size make it a promising solution for object detection.

**Figure 7:**
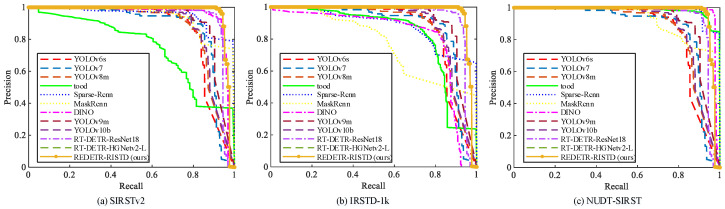
PR curves comparison between 11 detectors and the REDETR-RISTD model on three infrared small target detection datasets (SIRSTv2, IRSTD-1k, and NUDT-SIRST).

**Figure 8:**
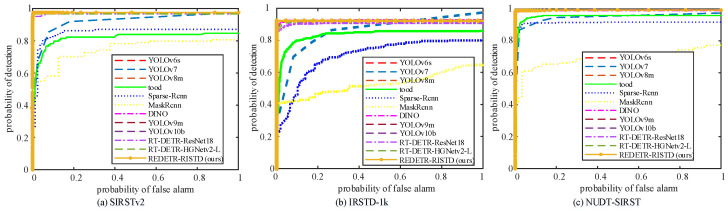
ROC curves comparison between 11 detectors and the REDETR-RISTD model on three infrared small target detection datasets (SIRSTv2, IRSTD-1k, and NUDT-SIRST).

Figure 7 and Figure 8 show the PR and ROC curves of the three methods on the three datasets, respectively. The following conclusions can be drawn from the curve charts: (1) REDETR-RISTD obtains the best results relatively quickly on all three datasets. (2) REDETR-RISTD achieves the best balance between model parameters, precision, and recall on all three datasets. (3) On all three datasets, REDETR-RISTD can correctly detect more infrared small targets with less computational power.

#### 4.4.2. Qualitative Analysis

To evaluate the performance of different detection methods for infrared small target detection, green and orange boxes are used in the visualization results to represent correct detections and abnormal detections, respectively.

Because the red boxes obtained in different scenes are small and the confidence scores are not clearly visible, green boxes and orange boxes are used instead to indicate correct detections and false or abnormal detections.

The number of green boxes reflects the number of correctly detected targets, while the number of orange boxes represents the number of false or missed detections.

In addition, the number inside the green box indicates the detection confidence, and the number inside the orange box indicates the false detection confidence. Red boxes are used to enclose the target areas.

**Figure 9:**
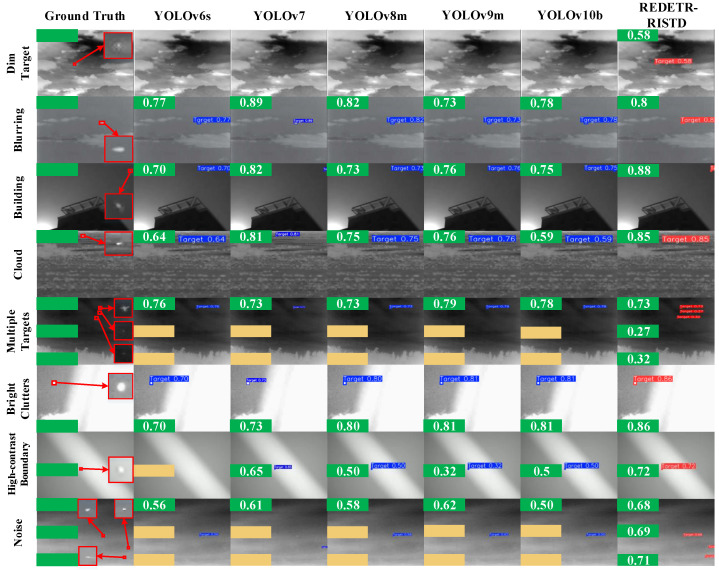
Comparison results of the detection performance of the YOLO series methods. On the far left, the ground truth provides locally zoomed-in annotations of infrared small targets. The first to the last rows show the results for detecting infrared small targets with dark appearances, blurred backgrounds, complex architectural masks, cloudy scenes, multiple targets, bright clutter, high-contrast boundaries, and high noise levels, respectively. Green bounding boxes denote true positive (TP) detection results, with numerical annotations indicating confidence scores; orange bounding boxes characterize anomalous detection events: those containing numerical values represent false positive (FP) predictions, where values reflect the confidence level of false detection, while unnumbered orange boxes indicate missing detection instances.

**Figure 10:**
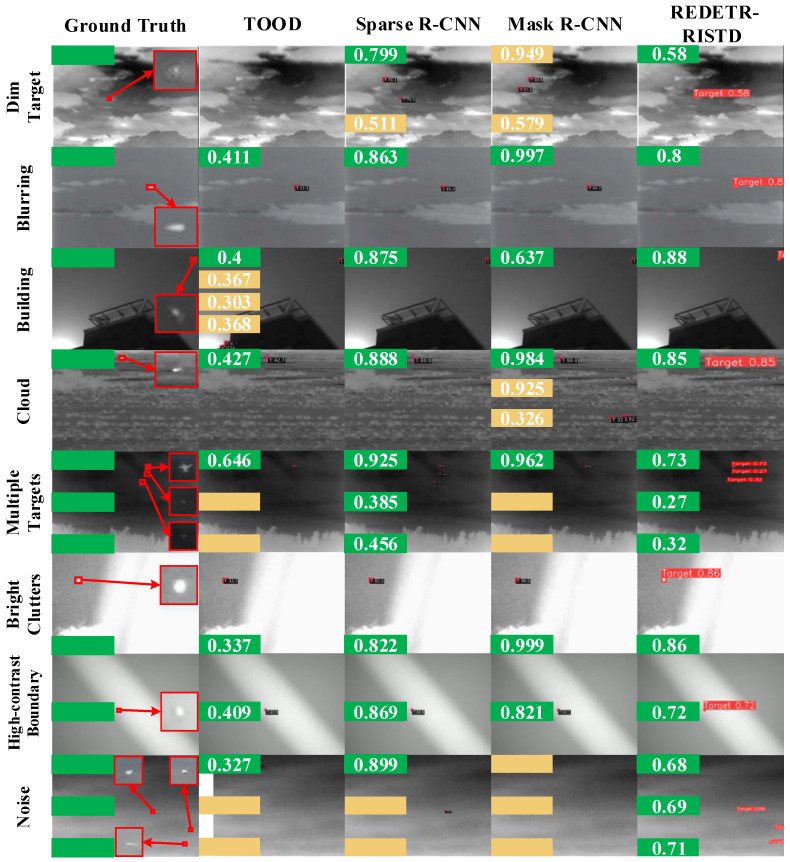
Comparison results of the detection performance of the typical one-stage and two-stage methods. On the far left, the ground truth provides locally zoomed-in annotations of infrared small targets. The first to the last rows show the results for detecting infrared small targets with dark appearances, blurred backgrounds, complex architectural masks, cloudy scenes, multiple targets, bright clutter, high-contrast boundaries, and high noise levels, respectively. Green bounding boxes denote true positive (TP) detection results, with numerical annotations indicating confidence scores; orange bounding boxes characterize anomalous detection events: those containing numerical values represent false positive (FP) predictions, where values reflect the confidence level of false detection, while unnumbered orange boxes indicate missing detection instances.

**Figure 11:**
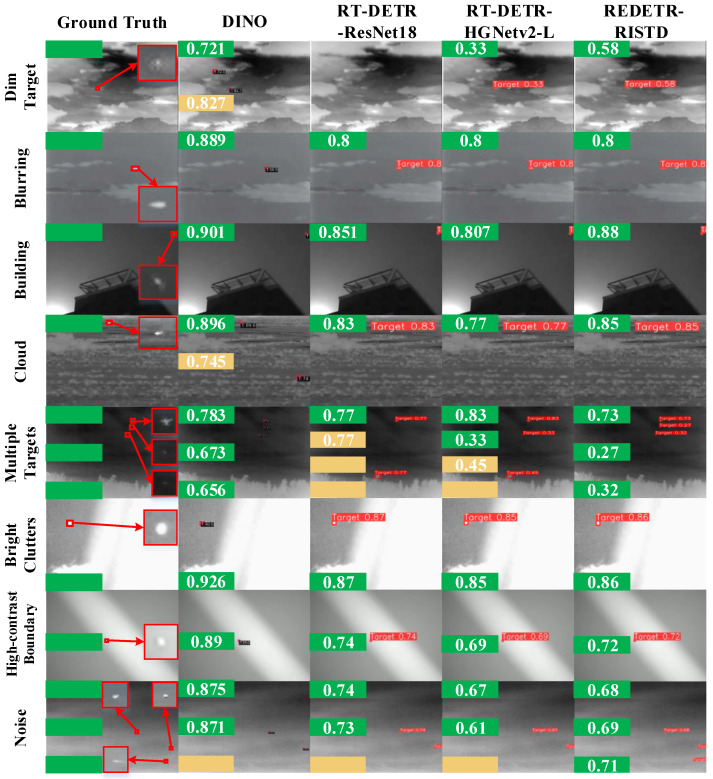
Comparison results of the detection performance of the transformer-based methods. On the far left, the ground truth provides locally zoomed-in annotations of infrared small targets. The first to the last rows show the results for detecting infrared small targets with dark appearances, blurred backgrounds, complex architectural masks, cloudy scenes, multiple targets, bright clutter, high-contrast boundaries, and high noise levels, respectively. Green bounding boxes denote true positive (TP) detection results, with numerical annotations indicating confidence scores; orange bounding boxes characterize anomalous detection events: those containing numerical values represent false positive (FP) predictions, where values reflect the confidence level of false detection, while unnumbered orange boxes indicate missing detection instances.

The visualization results are shown in Figure 9, Figure 10 and Figure 11. The left side of each figure displays the ground-truth target boxes in eight typical scenes. These scenes include dark appearance, blurred background, complex architectural occlusion, cloudy scene, multi-target, bright clutter, high-contrast boundary, and high noise.

Figure 9 shows the ground-truth bounding boxes of targets in eight scenarios on the left and the detection results of YOLO series and REDETR-RISTD on the right.

In the dark-appearance scenario, the target brightness is extremely low. The YOLO series methods failed to detect the target and did not show any detection boxes. In contrast, REDETR-RISTD successfully detected the target with a confidence of 58%. This success is due to its reparameterized multi-scale feature extraction module, which effectively extracts target features in low-signal-to-noise and low-contrast conditions.

In the blurred-background scenario, all methods detected the single target. However, detection confidence varied. YOLOv7 achieved a confidence of 89%, while REDETR-RISTD maintained around 80%. This indicates that REDETR-RISTD has high stability in blurred backgrounds. The attention-guided intra-scale contextual feature interaction module effectively suppresses background interference.

In the complex architectural occlusion scenario, REDETR-RISTD achieved a detection confidence of 88%. This value is significantly higher than the YOLO series methods, which ranged from 70% to 82%. This improvement is mainly attributed to the AICFI module, which optimizes the feature differences between the target and occluded regions. This reduces the impact of building occlusion and enables accurate detection of partially occluded targets.

In the cloudy scenario, REDETR-RISTD reached a detection confidence of 85%, which is clearly higher than YOLOv9m’s 76%. Its advantage comes from the RMSFE module’s multi-scale feature fusion capability. Under cloud interference, this module enhances the expression of target edge features and improves the detection of small targets.

In the multi-target scenario, the YOLO series methods only detected the largest target. They failed to recognize the other two targets. In contrast, REDETR-RISTD successfully detected all three targets with detection confidences of 0.73, 0.27, and 0.32. This demonstrates that the multi-scale pyramid feature fusion architecture, through a bidirectional fusion mechanism, effectively integrates target features at different scales to achieve comprehensive detection.

In the bright-clutter scenario, REDETR-RISTD reached a detection confidence of 86%. This shows strong anti-interference capability. It maintains high detection accuracy in complex backgrounds and effectively avoids false detections.

In the high-contrast-boundary scenario, YOLOv6s failed to detect the target, and other YOLO methods showed low detection confidence. In contrast, REDETR-RISTD successfully detected the target with a confidence of 72%. This indicates that it has strong target localization ability in high-contrast environments and can overcome the influence of high-contrast noise.

Finally, in the high-noise scenario, the YOLO series methods only detected the largest target. REDETR-RISTD, however, successfully detected three targets with detection confidences of 0.68, 0.69, and 0.71. This further proves its strong robustness in noisy environments and its ability to maintain high detection stability under complex interference conditions.

Figure 10 shows the ground-truth bounding boxes of targets in eight scenarios on the left and the detection results of TOOD, Sparse R-CNN, Mask R-CNN, and REDETR-RISTD on the right.

In the dark-appearance scenario, TOOD failed to detect the target. Sparse R-CNN detected the target with a confidence of 0.799 and produced one false positive with a confidence of 0.511. Mask R-CNN did not detect the target and generated two false positives with confidences of 0.949 and 0.579. In contrast, REDETR-RISTD successfully detected the target with a confidence of 58%. This result demonstrates that REDETR-RISTD, with its reparameterized multi-scale feature extraction module, can better capture target features in low-signal-to-noise and low-contrast environments.

In the blurred-background scenario, all four methods successfully detected the single target, with detection confidences of 41.1%, 86.3%, 99.7%, and 80%, respectively. REDETR-RISTD uses an attention-guided intra-scale contextual feature interaction module to effectively reduce background interference and ensure stable detection performance.

In the complex architectural occlusion scenario, TOOD detected the target with a confidence of 40% and produced three false positives with confidences of 0.367, 0.303, and 0.368. Sparse R-CNN, Mask R-CNN, and REDETR-RISTD detected the target with confidences of 87.5%, 63.7%, and 88%, respectively. The multi-scale feature fusion module of REDETR-RISTD is particularly effective in this scenario. It reduces the negative impact of building occlusion on detection results.

In the cloudy scenario, all methods successfully detected the target with detection confidences of 42.7%, 88.8%, 98.4%, and 85%, respectively. Mask R-CNN produced two false positives with confidences of 0.925 and 0.326. REDETR-RISTD further enhances the expression of target edge features through its multi-scale fusion mechanism, thus improving detection accuracy.

In the multi-target scenario, TOOD detected only the largest target with a confidence of 0.646 and missed two targets. Sparse R-CNN detected three targets with confidences of 0.925, 0.385, and 0.456. Mask R-CNN detected one target with a confidence of 0.962 and missed two targets. In contrast, REDETR-RISTD successfully detected three targets with confidences of 0.73, 0.27, and 0.32. Its multi-scale pyramid feature fusion and bidirectional interaction mechanism effectively integrate target features at different scales, achieving comprehensive target detection.

In the high-contrast-boundary scenario, the detection confidences were 33.7%, 82.2%, 99.9%, and 86% for TOOD, Sparse R-CNN, Mask R-CNN, and REDETR-RISTD, respectively. These results indicate that REDETR-RISTD can maintain high detection accuracy under complex background conditions.

Finally, in the high-noise scenario, TOOD detected one target with a confidence of 0.327 and missed two targets. Sparse R-CNN detected one target with a confidence of 0.899 and missed two targets. Mask R-CNN failed to detect the target. In contrast, REDETR-RISTD successfully detected three targets with confidences of 0.68, 0.69, and 0.71. Its multi-scale fusion and self-attention mechanism effectively reduce the impact of noise on feature extraction, thereby maintaining high detection performance even in high-noise conditions.

Figure 11 shows the visualization results of infrared small target detection in eight typical scenarios. The leftmost part displays the ground-truth bounding boxes of the targets, while the detection outputs of DINO, RT-DETR-ResNet18, RT-DETR-HGNet-L, and REDETR-RISTD are shown from left to right.

In the dark-appearance scenario, DINO correctly detected the target with a confidence of 0.721 but also produced a false positive with a confidence of 0.827. This indicates that it is susceptible to background noise under low-light conditions. RT-DETR-ResNet18 failed to detect the target, while RT-DETR-HGNet-L only detected the target with a confidence of 0.33, showing significantly inadequate performance. In contrast, REDETR-RISTD successfully detected the target with a confidence of 58%, which can be attributed to its reparameterized multi-scale feature extraction module, which is highly sensitive to weak target features in low-signal-to-noise environments.

In the blurred-background scenario, all methods successfully detected the single target, with detection confidences of 88.9% for DINO and 80% for RT-DETR-ResNet18, RT-DETR-HGNet-L, and REDETR-RISTD. This result suggests that when the target is well distinguished from the background, all models can perform well, and the attention-guided contextual feature interaction module of REDETR-RISTD plays a crucial role in maintaining stable detection performance.

In the complex architectural occlusion scenario, DINO detected the target with a confidence of 90.1%, while RT-DETR-ResNet18 and RT-DETR-HGNet-L achieved confidences of 85.1% and 80.7%, respectively. REDETR-RISTD reached 88%. The higher confidence of DINO indicates that it can extract stronger features when dealing with partial occlusion. However, REDETR-RISTD effectively mitigated the interference caused by occlusion through multi-scale feature fusion, ensuring accurate detection.

In the cloudy scenario, DINO detected the target with a confidence of 89.6%, but also produced a false positive with a confidence of 0.745. RT-DETR-ResNet18 and REDETR-RISTD achieved confidences of 83% and 85%, respectively, while RT-DETR-HGNet-L only reached 77%. Although DINO had a high detection confidence, its false positives show that it tends to capture erroneous features under cloud interference. REDETR-RISTD, on the other hand, uses its reparameterized structure to suppress interference information, resulting in more robust detection.

In the multi-target scenario, DINO detected all three targets with confidences of 0.783, 0.673, and 0.656, demonstrating an advantage when there are more targets. RT-DETR-ResNet18 detected only one target and missed two, while RT-DETR-HGNet-L detected two targets but produced one false positive and one missed target. In contrast, REDETR-RISTD successfully detected all three targets with confidences of 0.73, 0.27, and 0.32. This result confirms the effectiveness of REDETR-RISTD’s multi-scale pyramid feature fusion and bidirectional interaction mechanism in integrating features from different scales to handle multi-target detection tasks comprehensively.

In the bright-clutter scenario, all methods exhibited high detection confidences, with DINO reaching 92.6%, RT-DETR-ResNet18 at 87%, RT-DETR-HGNet-L at 85%, and REDETR-RISTD at 86%. This shows that all methods can recognize the target in a bright and cluttered background, with REDETR-RISTD maintaining a relatively stable performance.

For the high-contrast-boundary scenario, DINO achieved a detection confidence of 89%, showing strong target localization capability. In comparison, RT-DETR-ResNet18, RT-DETR-HGNet-L, and REDETR-RISTD achieved confidences of 74%, 69%, and 72%, respectively. Although DINO performed relatively well under these conditions, REDETR-RISTD effectively reduced the negative impact of high-contrast noise through its adaptive noise suppression mechanism, ensuring stable detection results.

In the high-noise scenario, DINO detected two targets with confidences of 0.875 and 0.871, but missed one target. RT-DETR-ResNet18 and RT-DETR-HGNet-L also detected two targets, but both missed one target, with confidences ranging from 0.74 to 0.73 and 0.67 to 0.61, respectively. In contrast, REDETR-RISTD detected three targets with confidences of 0.68, 0.69, and 0.71, demonstrating stronger noise suppression ability. This indicates that in noisy environments, traditional methods are prone to interference in feature extraction, while REDETR-RISTD’s multi-scale feature fusion and self-attention mechanism effectively alleviated this issue, ensuring comprehensive target detection.

### 4.5. Ablation Experiment

REDETR-RISTD contains three key components: the backbone RepEMSNet, the intra-scale contextual feature interaction AICFI, and the feature fusion framework MSPFN, which compose a model capable of accurately detecting infrared small targets. Ablation experiments were conducted to compare with the baseline model RT-DETR-ResNet18 to validate the effectiveness of the proposed modules, and the ablation experiments included six different combinations.

Group 1: RT-DETR-ResNet18 (Baseline).

Group 2: RT-DETR+RepEMSNet.

Group 3: RT-DETR+RepEMSNet+AICFI.

Group 4: RT-DETR+RepEMSNet+AICFI +MSPFN.

Group 5: REDETR-RISTD (Ours).

**Figure 12:**
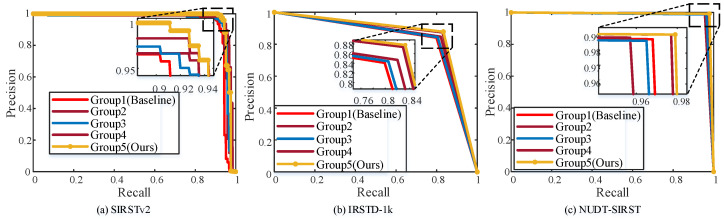
PR curve of the different groups on three infrared small target detection datasets (SIRSTv2, IRSTD-1k, and NUDT-SIRST).

The performance of each module is qualitatively analyzed by plotting the PR curves of the five combination methods. As shown in Figure 12, compared with the baseline model, the PR curves of the proposed modules’ combinations Group 2, Group 3, Group 4, and Group 5 have a larger area enclosed with the horizontal and vertical axes than that of Group 1, which fully proves the effectiveness of the three proposed modules.

Table 4 demonstrates the detection results, where Group 1, which is absent from all three modules, represents the baseline model RT-DETR-ResNet18, and Group 1–Group 4 all use GIoU.

As shown in Table 4, the proposed method exhibits an increase in recall, AP50, and AP50-95, and a decrease in the number of parameters compared to the baseline model, achieving an overall improvement in the detector’s performance.

Specifically, compared to the baseline combination Group 1, after replacing the RepEMSNet backbone, the model parameters in Group 2 decreased by 30.3% and 21.97%, respectively, and the detection accuracy metrics, AP50 and AP50-95, increased to 95% and 47.8%, respectively.

In Group 3, after adding the AICFI module, AP50-95 is improved to 49.6%, and the number of model parameters is further reduced to 13.811 M.

After combining the first three modules, Group 4 is obtained. Currently, AP50 reaches 96.4%, recall is improved to 92.6%, and the number of parameters is reduced by 30.3%, respectively.

Finally, the original GIoU is replaced by EnIoU, which accelerates the network’s convergence while increasing AP50-95 to 51.6%. At this time, the number of parameters and computations are further reduced by 30.5% and 19.5%, respectively, compared to the baseline model.

The significant change in AP50-95 is caused by the small number of pixels occupied by the small targets, resulting in a small area covered by the genuine bounding box. Slight deviations occurring in the prediction bounding box can also change significantly with the IoU of the GT box. Using AICFI to interact with the deep feature map output from RepEMSNet with in-scale features makes the network more sensitive to the location information of small targets. The EnIoU is also utilized to focus on the degree and location of overlap between the prediction frame and the GT box to accelerate the network’s convergence and generate a prediction bounding box consistent with the GT box, thus improving AP50-95.

To validate the performance of the proposed backbone model in this thesis for detecting infrared small targets, we conducted a comprehensive comparison with nine current advanced backbone models such as ResNet, Transformer, etc., and the comparison results are shown in Table 5.

In Table 5, combining the four metrics of recall, AP50, AP50-95 and Parameters, only the proposed RepEMSNet backbone performs in the top four, realizing an effective balance between detection accuracy, parameter count, and computational cost. Compared to the latest StarNet backbone, RepEMSNet’s recall, AP50, and AP50-95 are improved by 5.9%, 4.7%, and 2.6%, respectively. The number of parameters is reduced by 1.944. Compared to ResNet-50 and CSwinTransformer, recall, AP50, and AP50-95 are improved by 5.9%, 4.7%, and 2.6%, respectively. AP50 and AP50-95 metrics decreased by about 1%, but Parameters decreased by 28.111 M and 16.64 M.

### 4.6. Impacts of Reparameterized Structures

The proposed RepEMSNet backbone network employs a structural reparameterization operation, which converts a multi-branch structure into a single-branch structure and integrates the batch normalization operation into the convolution operation. These operations can effectively reduce memory occupancy and improve computational efficiency without affecting detection accuracy.

Table 6 demonstrates the change in REDETR-RISTD inference time before and after structural reparameterization. The batch size and input image resolution are 16 and 640 × 640 pixels, respectively. The results show that the overall inference time of the proposed model is reduced by 21% on CPU and 15.1% on GPU by structural reparameterization.

### 4.7. Loss Function Comparison Experiment

To verify the effectiveness of the proposed EnIoU loss function, we analyzed it in comparison with existing loss functions (e.g., GIoU [60], DIoU, CIoU [61], EIoU [62], SIoU [63], Inner-IoU [64], and MPDIoU [65]). The auxiliary bounding box was resized and tested using various scale settings. The data in Table 7 show that the model equipped with the EnIO loss function at a scale of ratio = 0.7 exhibits higher detection accuracy. Compared to the baseline model with the GIoU loss function, the recall metric improved by 0.1%, AP50 by 0.1%, and AP50-95 by 3.1%. The results suggest that using the EnIoU loss function may lead to more consistent bounding box regression and improved prediction accuracy.

To clearly illustrate the detection performance of models trained with different IoU loss functions, we use radar charts to display the four metrics P, R, AP50, and AP50-95. To ensure the metrics are comparable, we normalize them. This allows us to present the performance of the models in a unified coordinate system.

**Figure 13:**
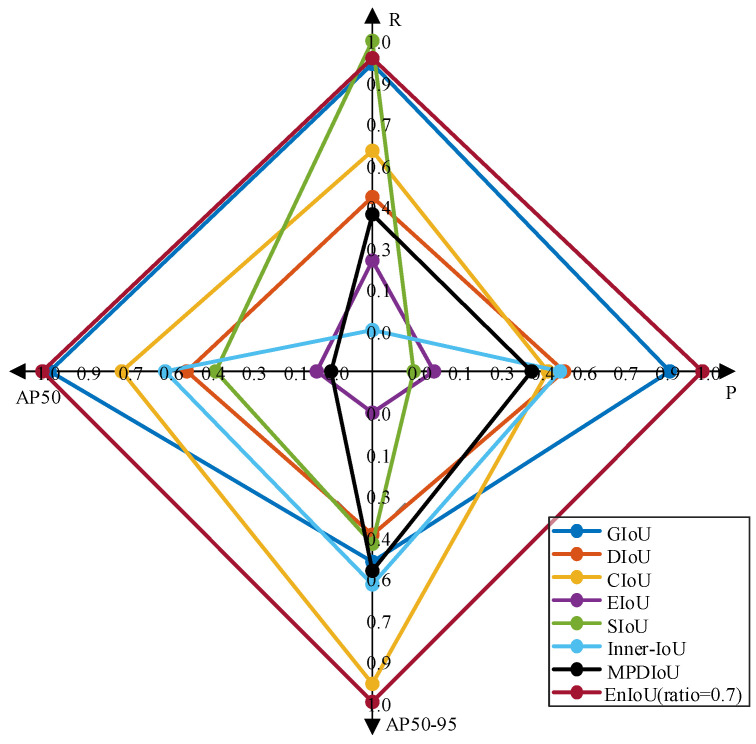
Performance comparison of eight IoU loss functions under the metrics P, R, AP50, and AP50-95.

From Figure 13, it can be observed that when EnIoU is used as the bounding box loss function with the hyperparameter ratio = 0.7, the radar chart exhibits the largest coverage area. This result indicates superior performance in P, R, AP50, and AP50-95, demonstrating the strongest overall detection capability. The larger polygon area reflects not only higher values in individual metrics but also a balanced improvement in multi-metric performance. The superior performance is attributed to two key factors. First, EnIoU achieves coordinated optimization by addressing sample differentiation, positional accuracy, and multi-scale feature integration through its adaptive weighting mechanism. It effectively balances the trade-off between precision and recall. Second, EnIoU leverages geometric constraints and multi-scale feature fusion to enhance adaptability to complex scenarios, ensuring robust detection performance.

**Figure 14:**
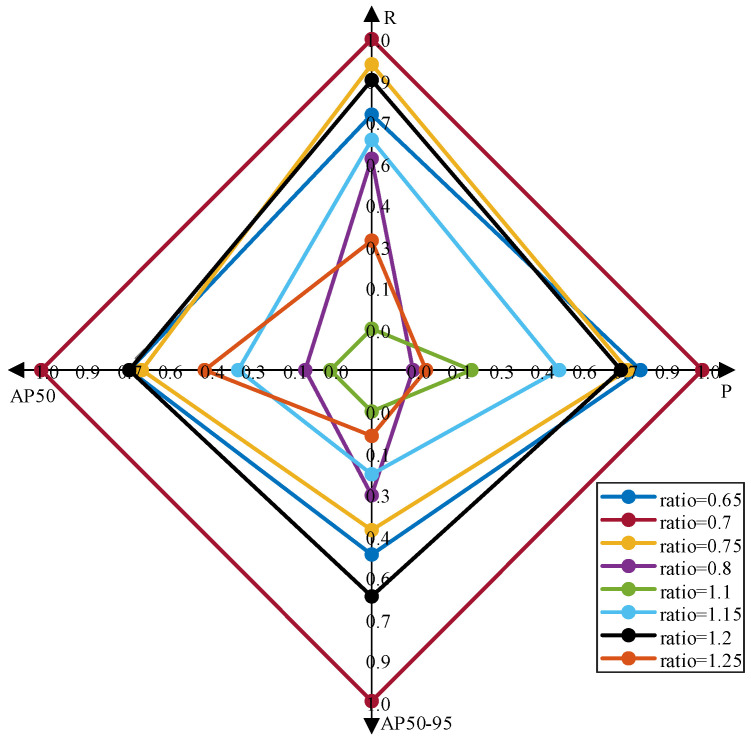
Performance comparison of the EnIoU loss function with eight different hyperparameter ratio settings under the metrics P, R, AP50, and AP50-95.

Furthermore, Figure 14 demonstrates the training results of EnIoU under different hyperparameter ratio settings. It is evident that variations in ratio lead to significant fluctuations in model performance across metrics. Among these configurations, the radar chart corresponding to ratio = 0.7 exhibits the largest coverage area. This observation further validates the advantage of this parameter configuration in balancing loss weighting and gradient feedback, ensuring optimal training dynamics.

**Figure 15:**
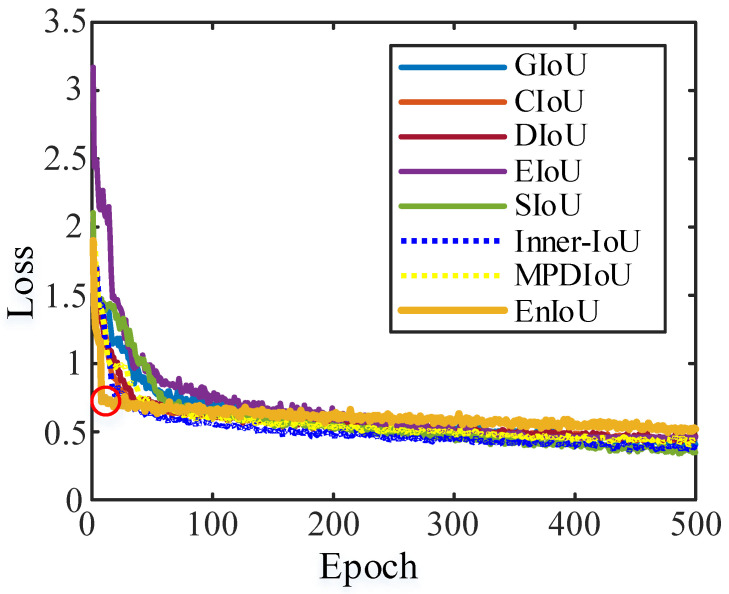
Convergence curves of seven typical IoU and EnIoU loss functions. The red circle indicates the convergence point of the proposed EnIoU training.

To analyze the speed of convergence of model training under different IoU, we show the convergence curves of the eight loss functions in Figure 15. All loss functions eventually converge, yet EnIoU achieves a stable state in fewer epochs. The red circle in Figure 15 marks the convergence epoch. This rapid convergence is attributed to the design of EnIoU. It adopts an adaptive loss weighting mechanism that adjusts the size of the auxiliary bounding box based on the sample’s IoU, using a smaller box for high-IoU samples and a larger box for low-IoU samples. In addition, EnIoU improves positional accuracy by calculating the Euclidean distance between the vertices of the predicted and GT boxes, which ensures closer alignment. The design further incorporates multi-scale feature fusion to enhance the model’s adaptability to targets of different sizes. Experimental results confirm that this design not only speeds up convergence but also improves overall detection performance.

### 4.8. Comparative Experiment of Bounding Box-Based Detection Method and Segmentation-Based Method

In this section, we present a comprehensive comparison between the bounding box-based detection method and segmentation-based approaches for infrared small target detection. We first offer a quantitative evaluation of performance metrics across multiple challenging datasets, and then provide qualitative visualizations that further illustrate detection outcomes under diverse conditions. The following results, including the performance table and detection visualizations, demonstrate the robustness and superiority of our method over traditional segmentation techniques.

From Table 8, it is clear that the infrared small target detection method based on bounding box detection (REDETR-RISTD) significantly outperforms the six segmentation-based methods on multiple metrics. First, REDETR-RISTD achieves the highest performance in three core metrics: precision (P), recall (R), and detection probability (Pd) across the SIRSTv2, IRSTD-1k, and NUDT-SIRST datasets. For example, on the SIRSTv2 dataset, the method achieves a precision of 0.991, a recall of 0.927, and a detection probability of 0.976. The values of other methods are much lower. This consistent advantage shows that the bounding box method is more accurate and robust in locating small targets and capturing their features.

On the other hand, segmentation-based methods depend on pixel-level segmentation. They are easily affected by background noise and blurred target details. As a result, their precision and recall are lower than those of the bounding box method. Although REDETR-RISTD shows a slightly higher false alarm rate (Fa) on some datasets, its false alarm rate is only 1.25 on the NUDT-SIRST dataset. This is a clear advantage compared to other methods. In infrared small target detection, high precision and high recall are critical. A slight increase in the false alarm rate can be remedied by further optimization and threshold adjustment.

Furthermore, REDETR-RISTD demonstrates strong generalization ability across different datasets, which further proves its robustness. Whether it is the complex background of the SIRSTv2 dataset or other scenarios, the method consistently delivers high detection performance. This advantage shows that the bounding box detection method can effectively overcome the shortcomings of traditional segmentation methods, such as loss of detail and blurred target localization, and thus achieves more accurate detection results.

**Figure 16:**
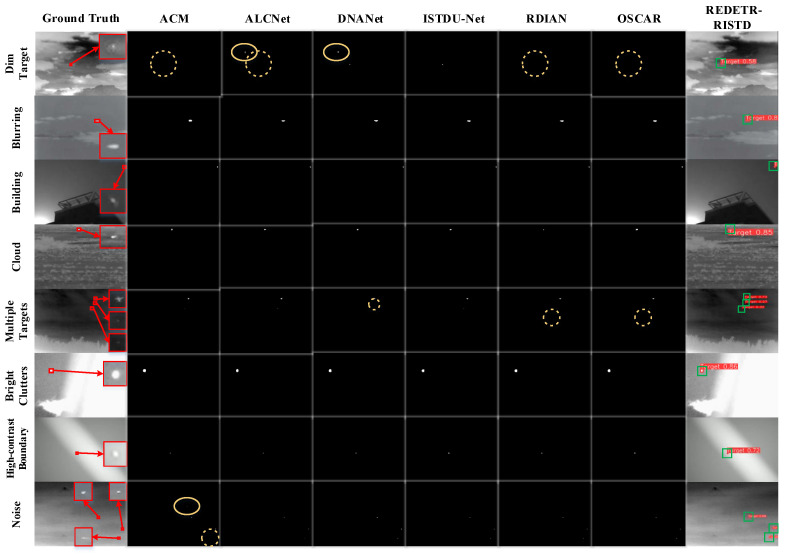
The detection results of segmentation-based methods and REDETR-RISTD across eight different scenarios are presented. Dashed circles indicate missed detections, while solid ellipses mark false detections. The green box indicates that the target has been detected correctly.

Based on the visual results in Figure 16, we discuss the performance of each method in different scenarios in sequence. First, the ACM method fails to detect the target in low-brightness (dim target) images. In the Noise image with three targets and significant background noise, it only correctly detects two targets, showing false positives and false negatives. This indicates that the ACM method is sensitive to low contrast and noise interference. Next, ALCNet also has false positives and false negatives in low-brightness images. Although it detects all targets in other scenarios, its robustness is limited under challenging conditions. DNANet shows a false positive in low-brightness images and only detects one target correctly. It also misses one target in the multi-target scenario but performs reasonably well in other situations. In contrast, ISTDU-Net detects all targets across all scenarios, demonstrating good adaptability to complex backgrounds and low-contrast conditions. Furthermore, both RDIAN and U-Net fail to detect the target in low-brightness images and miss one target in the multi-target scenario. While their performance is generally good in other conditions, their overall stability is weaker. Finally, our proposed bounding box-based detection method, REDETR-RISTD, accurately detects targets in all scenarios, whether in low brightness, complex backgrounds, or multi-target conditions, with no false positives or false negatives.

Overall, traditional segmentation methods are prone to be affected by background noise and blurred target details in low-brightness and complex scenarios, leading to unstable detection results. In contrast, the REDETR-RISTD bounding box-based method shows higher robustness and accuracy. These results confirm the advantages of using bounding box strategies in infrared small target detection tasks, providing a more reliable solution for practical applications.

## 5. Conclusions

We propose REDETR-RISTD, an infrared small target detection network with a reparameterizable backbone and multi-scale feature fusion. We introduce a reparameterizable backbone called RepEMSNet that speeds up inference, improves accuracy, and gradually enlarges the sensory field using stacked 3×3 convolutions. In the encoder, we use AICFI to capture local details and global dependencies in the multi-scale feature maps, enabling the network to focus on salient features for infrared small target detection. We also developed a new framework named MSPFN to efficiently fuse multi-scale features using bottom-up fusion and top-down path enhancement strategies. Three modules were designed to extract both local and global contextual information and to significantly enhance feature diversity and expressive capability. Experimental results show that our network outperforms other advanced detectors, and ablation experiments confirm the effectiveness of RepEMSNet, AICFI, and MSPFN. Given its enhanced accuracy and efficiency in detecting small, dim targets against complex backgrounds, REDETR-RISTD demonstrates significant potential for real-world applications, particularly in demanding fields such as infrared search-and-rescue missions and various aerial or ground-based monitoring scenarios where timely and reliable detection is critical.

Infrared small target detection still faces challenges such as background occlusion, interference, and camouflage spoofing, and it is evolving toward infrared tracking missions. In the future, we will further optimize the REDETR-RISTD architecture by exploring improved backbone and feature fusion methodologies to reduce parameters and computation while enhancing accuracy and speed. We will also investigate interpretable learning combined with spatio-temporal information and apply these advances to infrared small target tracking.

## Figures and Tables

**Table 1:** Configurations of RepEMSNet. The original input infrared image resolution is 640 × 640 pixels.

Module	Number	Scaling Factor	Output Resolution (Pixels)	Output Channel	Output Feature Map
Conv1	1	-	320 × 320	64	S1
Conv1	1	-	160 × 160	128	-
RMSFE	1	s=0.5	160 × 160	128	S2
Conv1	1	-	80 × 80	256	-
RMSFE	1	s=0.5	80 × 80	256	S3
Conv1	1	-	40 × 40	384	-
RMSFE	1	s=1	40 × 40	384	S4
Conv1	1	-	20 × 20	384	-
RMSFE	3	s=1	20 × 20	384	S5

**Table 2:** Experimental hardware parameters.

Name	Configuration
Operating system	Win11
Computing platform	CUDA 11.7
CPU	Intel Core i5
GPU	NVIDIA GeForce RTX 3060 (GALAX Technology, Hong Kong, China)
GPU memory size	12 G

**Table 3:** Quantitative analysis results. The best-performing method is presented in bold, and the second-best method is shown in bold italics.

Methods	Dataset												
	SIRSTv2				IRSTD-1k				NUDT-SIRST				Parameters/M
	P	R	AP50	AP50-95	P	R	AP50	AP50-95	P	R	AP50	AP50-95	
YOLOv6s	0.906	0.776	0.864	0.446	0.831	0.721	0.79	0.359	0.904	0.941	0.965	0.754	16.298
YOLOv7	0.89	0.847	0.876	0.407	0.827	0.718	0.759	0.329	0.959	0.915	0.94	0.623	**6.195**
YOLOv8m	0.926	0.758	0.875	0.447	0.883	0.721	0.809	0.373	0.977	0.904	0.968	0.766	25.84
TOOD	0.689	0.661	0.704	0.268	0.839	0.745	0.809	0.363	0.952	0.925	0.958	0.696	32.018
Sparse R-CNN	0.897	0.863	0.888	0.477	0.826	0.743	0.81	0.384	0.986	0.91	0.944	0.649	77.8
Mask R-CNN	0.923	0.79	0.888	0.511	0.807	0.561	0.691	0.357	0.811	0.814	0.877	0.563	43.991
DINO	0.927	0.923	0.948	0.485	0.836	0.816	0.825	0.348	0.983	0.964	0.978	0.771	47.54
YOLOv9m	0.918	0.812	0.917	0.472	0.87	0.735	0.808	0.37	0.922	0.891	0.963	0.769	20.156
YOLOv10b	0.954	0.774	0.886	0.487	0.776	0.728	0.807	0.381	0.97	0.915	0.978	0.668	19.005
RT-DETR-ResNet18	0.958	0.911	0.94	0.468	0.841	0.799	0.827	0.37	0.989	0.968	***0.99***	0.724	19.873
RT-DETR-HGNetv2-L	0.974	0.918	0.943	0.477	0.857	0.784	0.841	0.381	**0.995**	**0.986**	**0.995**	**0.809**	31.986
REDETR-RISTD (Ours)	**0.991**	**0.927**	**0.965**	**0.516**	**0.878**	**0.833**	**0.843**	**0.388**	***0.992***	***0.979***	***0.985***	***0.786***	***13.814***

**Table 4:** Detection results for different modules. ✓ and × indicate the presence or absence of the corresponding module, respectively.

Group	RepEMSNet	AICFI	MSPFN	EnIoU	R	AP50	AP50-95	Parameters/M
1	×	×	×	×	0.911	0.94	0.468	19.873
2	✓	×	×	×	0.929	0.95	0.478	13.845
3	✓	✓	×	×	0.918	0.947	0.496	13.811
4	✓	✓	✓	×	0.926	0.964	0.485	13.851
5	✓	✓	✓	✓	0.927	0.965	0.516	13.814

**Table 5:** Experimental results of different backbones. The best and the fourth-best values are denoted in bold, respectively.

Backbones	R	AP50	AP50-95	Parameters/M
ResNet-18	0.911	0.94	0.468	19.873
ResNet-34	0.887	**0.947**	**0.480**	31.106
ResNet-50	**0.935**	**0.951**	**0.493**	41.956
HGNet-V2	**0.918**	0.943	0.477	31.986
StarNet	0.870	0.903	0.452	**15.789**
EfficientViT	0.903	0.922	0.442	**10.703**
RepViT	0.887	0.907	0.424	**4.471**
CSwinTransformer	**0.932**	**0.948**	**0.483**	**30.485**
SwinTransformer	0.876	0.913	0.440	36.313
RepEMSNet	**0.929**	**0.950**	**0.478**	**13.845**

**Table 6:** Inference time on GPUs and CPUs. ✓ means adopting reparameterization, and × means not adopting reparameterization.

Reparameter	Device	Preprocess	Inference	Postprocess
×	CPU	1.0 ms	390.4 ms	0.1 ms
✓	CPU	1.2 ms	307.8 ms	0.1 ms
×	RTX3060	0.7 ms	13.1 ms	0.1 ms
✓	RTX3060	0.8 ms	10.8 ms	0.2 ms

**Table 7:** Results of the effect of different loss functions. The best values are denoted in bold.

Loss Function	P	R	AP50	AP50-95
GIoU	0.983	0.926	0.964	0.485
DIoU	0.957	0.903	0.945	0.479
CIoU	0.953	0.911	0.954	0.512
EIoU	0.925	0.892	0.927	0.452
SIoU	0.92	0.93	0.941	0.481
Inner-IoU	0.956	0.88	0.948	0.49
MPDIoU	0.949	0.9	0.925	0.487
EnIoU (ratio = 0.65)	0.972	0.903	0.944	0.474
EnIoU (ratio = 0.7)	**0.991**	**0.927**	**0.965**	**0.516**
EnIoU (ratio = 0.75)	0.968	0.919	0.941	0.467
EnIoU (ratio = 0.8)	0.902	0.889	0.902	0.457
EnIoU (ratio = 1.1)	0.92	0.835	0.896	0.433
EnIoU (ratio = 1.15)	0.947	0.895	0.918	0.451
EnIoU (ratio = 1.2)	0.966	0.914	0.944	0.486
EnIoU (ratio = 1.25)	0.906	0.863	0.926	0.44

**Table 8:** Our model vs. SOTA models: comparison of P, R, Pd, and Fa ($\times 10^{-5}$) values on the SIRSTv2, IRSTD-1k, and NUDT-SIRST datasets. The best performers are in bold.

Methods	Dataset											
	SIRSTv2				IRSTD-1k				NUDT-SIRST			
	P	R	Pd	Fa	P	R	Pd	Fa	P	R	Pd	Fa
ACM	0.721	0.777	0.879	5.85	0.679	0.757	**0.923**	5.93	0.706	0.869	0.971	2.2
ALCNet	0.838	0.665	0.902	1.84	0.7	0.82	0.913	6	0.809	0.797	0.963	1.29
DNANet	0.876	0.863	0.97	1.8	0.82	0.726	0.879	1.31	0.954	0.959	0.99	1.22
ISTDU-Net	0.852	0.796	0.962	2.15	0.78	0.77	0.899	3.51	0.947	0.941	0.987	1.28
RDIAN	0.899	0.72	0.939	6.26	0.828	0.67	0.866	0.67	0.917	0.882	0.978	1.29
OSCAR	0.873	0.742	0.909	4.2	0.769	0.76	0.896	2.83	0.9	0.927	0.974	1.35
REDETR-RISTD (Ours)	**0.991**	**0.927**	**0.976**	6.9	**0.922**	**0.833**	**0.922**	4.59	**0.992**	**0.979**	**0.992**	1.25

## Data Availability

The data underlying the findings of this study are available upon reasonable request from the corresponding author.
